# Genome-wide association mapping of black point reaction in common wheat (*Triticum aestivum* L.)

**DOI:** 10.1186/s12870-017-1167-3

**Published:** 2017-11-23

**Authors:** Jindong Liu, Zhonghu He, Awais Rasheed, Weie Wen, Jun Yan, Pingzhi Zhang, Yingxiu Wan, Yong Zhang, Chaojie Xie, Xianchun Xia

**Affiliations:** 1 Institute of Crop Sciences, National Wheat Improvement Center, Chinese Academy of Agricultural Sciences (CAAS), 12 Zhongguancun South Street, Beijing, 100081 China; 2 Department of Plant Genetics & Breeding/State Key Laboratory for Agrobiotechnology, China Agricultural University, 2 Yuanmingyuan West Road, Beijing, 100193 China; 3 International Maize and Wheat Improvement Center (CIMMYT) China Office, c/o CAAS, 12 Zhongguancun South Street, Beijing, 100081 China; 4 Institute of Cotton Research, Chinese Academy of Agricultural Sciences (CAAS), 38 Huanghe Street, Anyang, Henan 455000 China; 5 Crop Research Institute, Anhui Academy of Agricultural Sciences, 40 Nongke South Street, Hefei, Anhui 230001 China

**Keywords:** 660 K SNP array, 90 K SNP array, Enzymatic browning, Favorable and unfavorable allele, GWAS

## Abstract

**Background:**

Black point is a serious threat to wheat production and can be managed by host resistance. Marker-assisted selection (MAS) has the potential to accelerate genetic improvement of black point resistance in wheat breeding. We performed a genome-wide association study (GWAS) using the high-density wheat 90 K and 660 K single nucleotide polymorphism (SNP) assays to better understand the genetic basis of black point resistance and identify associated molecular markers.

**Results:**

Black point reactions were evaluated in 166 elite wheat cultivars in five environments. Twenty-five unique loci were identified on chromosomes 2A, 2B, 3A, 3B (2), 3D, 4B (2), 5A (3), 5B (3), 6A, 6B, 6D, 7A (5), 7B and 7D (2), respectively, explaining phenotypic variation ranging from 7.9 to 18.0%. The highest number of loci was detected in the A genome (11), followed by the B (10) and D (4) genomes. Among these, 13 were identified in two or more environments. Seven loci coincided with known genes or quantitative trait locus (QTL), whereas the other 18 were potentially novel loci. Linear regression showed a clear dependence of black point scores on the number of favorable alleles, suggesting that QTL pyramiding will be an effective approach to increase resistance. In silico analysis of sequences of resistance-associated SNPs identified 6 genes possibly involved in oxidase, signal transduction and stress resistance as candidate genes involved in black point reaction.

**Conclusion:**

SNP markers significantly associated with black point resistance and accessions with a larger number of resistance alleles can be used to further enhance black point resistance in breeding. This study provides new insights into the genetic architecture of black point reaction.

**Electronic supplementary material:**

The online version of this article (10.1186/s12870-017-1167-3) contains supplementary material, which is available to authorized users.

## Background

Black point, characterized by dark discoloration at the embryo end of kernels, occurs in most wheat growing regions of the world including China, USA, Australia, Canada and Serbia [1, 2]. It can downgrade end-use quality of the grain due to seed discoloration [3]. Many marketing authorities have regulations on the incidence of black point, such as ≤4% in the USA, ≤ 5% in Australia, and ≤10% in Canada [4], indicating that grain with black point symptoms is more difficult to market with consequent economic losses to producers. In addition, black point can decrease the germination percentage and cause impaired seedling development [4]. It can also lead to the presence of toxic secondary metabolites, such as Alternaria mycotoxin and Alternariol monomethyl ether [5–7] that may cause oesophageal cancer [8].

Many studies indicate that black point is enhanced by abiotic stresses, as symptoms more likely occur after exposure to high humidity and extreme temperatures during grain filling [9, 10]. However, the causes of black point remain unclear and contradictory. Fungi are considered as the causal agents of black point [1]; these include *Alternaria alternata* [5, 11], *Bipolaris sorokiniana* [12] and *Fusarium proliferatum* [6]. However, direct association between the presence of fungi and black point development has been discounted by some workers [13–15], who pointed out that it may be caused by enzymatic browning following stress. Oxidases, such as peroxidases (POD) [15, 16], polyphenol oxidase (PPO) [17, 18] and lipoxygenase (LOX) [19], that catalyze oxidation of phenolic compounds to brown or black pigments (melanins and quinines) [18, 20], may be triggered by high humidity during the later stages of grain filling. Susceptible varieties have higher POD [15, 21] and phenylalanine ammonia-lyase (PAL) (an enzyme involved in phenolic acid biosynthesis) [21] activities.

Although several cultural, biological and chemical control strategies have been used to control black point, breeding resistant cultivars remains the most effective, economic and environmentally sustainable approach to control this disease [4, 22, 23]. Previous studies on the known genetic basis of black point resistance involved classical linkage-mapping methods using bi-parental populations [22–24], in which only two allelic effects can be evaluated for any single locus. Recent advances in genomics, particularly development of the wheat 90 K [25], 660 K (JZ Jia, pers. comm.) and 820 K SNP arrays [26] have made it feasible to genotype large germplasm collections with high-density SNP markers. As a result, the GWAS based on linkage disequilibrium (LD) has been widely adopted to investigate existing allelic diversity for important and complex agronomic traits. Compared with classical linkage-mapping, GWAS permits a more representative gene pool and a higher mapping resolution, because all historical meiotic events that have occurred in the ancestors of a diverse germplasm panel can be used [27]. Moreover, GWAS bypasses the expense and time of developing mapping populations, and enables the mapping of many traits in one set of genotypes, making the method more efficient and less expensive than linkage mapping [28]. Thus, GWAS has become a powerful alternative approach for linkage mapping [29]. GWAS has been applied to investigate a range of traits, including disease resistance [30, 31], end-use quality [32], and yield components [33–35].

The Yellow and Huai River Valleys Facultative Wheat Region is one of the most important agricultural regions of wheat production in China with an area of 15.3 million hectares. Black point has become one of the important diseases in this region due to increased water management and fertilizer use. Breeding for black point resistance could be greatly improved by the identification and use of closely associated molecular markers. Although GWAS has become a powerful approach to dissect the genetic architecture for many traits, it has not been used to analyze traits related to black point. In the present study, we used a diverse panel of 166 elite wheat cultivars in GWAS to (1) dissect the genetic architecture of black point resistance, (2) identify SNPs significantly associated with black point resistance, and (3) search for candidate black point resistance genes for further study.

## Results

### Marker coverage and genetic diversity

A total of 18,920 SNPs from the 90 K and 283,652 from the 660 K SNP array based on the consensus genetic maps and physical map (IWGSC, http://www.wheatgenome.org/) were chosen for GWAS of black point reaction in 166 wheat cultivars (Additional file 1: Table S1). After removing the SNPs with minor allele frequency (MAF) < 5% (28,935 SNPs) and missing data >20% (13,715 SNPs), 259,922 SNPs were employed for subsequent analysis (Additional file 2: Table S2). These markers spanned a physical distance of 14,063.9 Mb, with an average density of 0.054 Mb per marker. Total of 89,519 (34.4%), 146,270 (56.3%) and 24,133 (9.3%) markers were from the A, B and D genomes, respectively, with corresponding map lengths of 4934.5, 5179.0 and 3950.4 Mb. The marker density for the D genome (0.202 Mb per marker) was lower than that for the A (0.099 Mb per marker) and B (0.042 Mb per marker) genomes. The average genetic diversity and polymorphism information content (PIC) for the whole genome were 0.356 (0.009–0.500) and 0.285 (0.009–0.380), respectively. Both the genetic diversity and PIC of the A (0.365 and 0.291) and B (0.363 and 0.289) genomes were higher than the D (0.340 and 0.265) genome. The number of markers, map length, genetic diversity and PIC for each chromosome are shown in Additional file 2: Table S2.

### Population structure and linkage disequilibrium

**Fig. 1:**
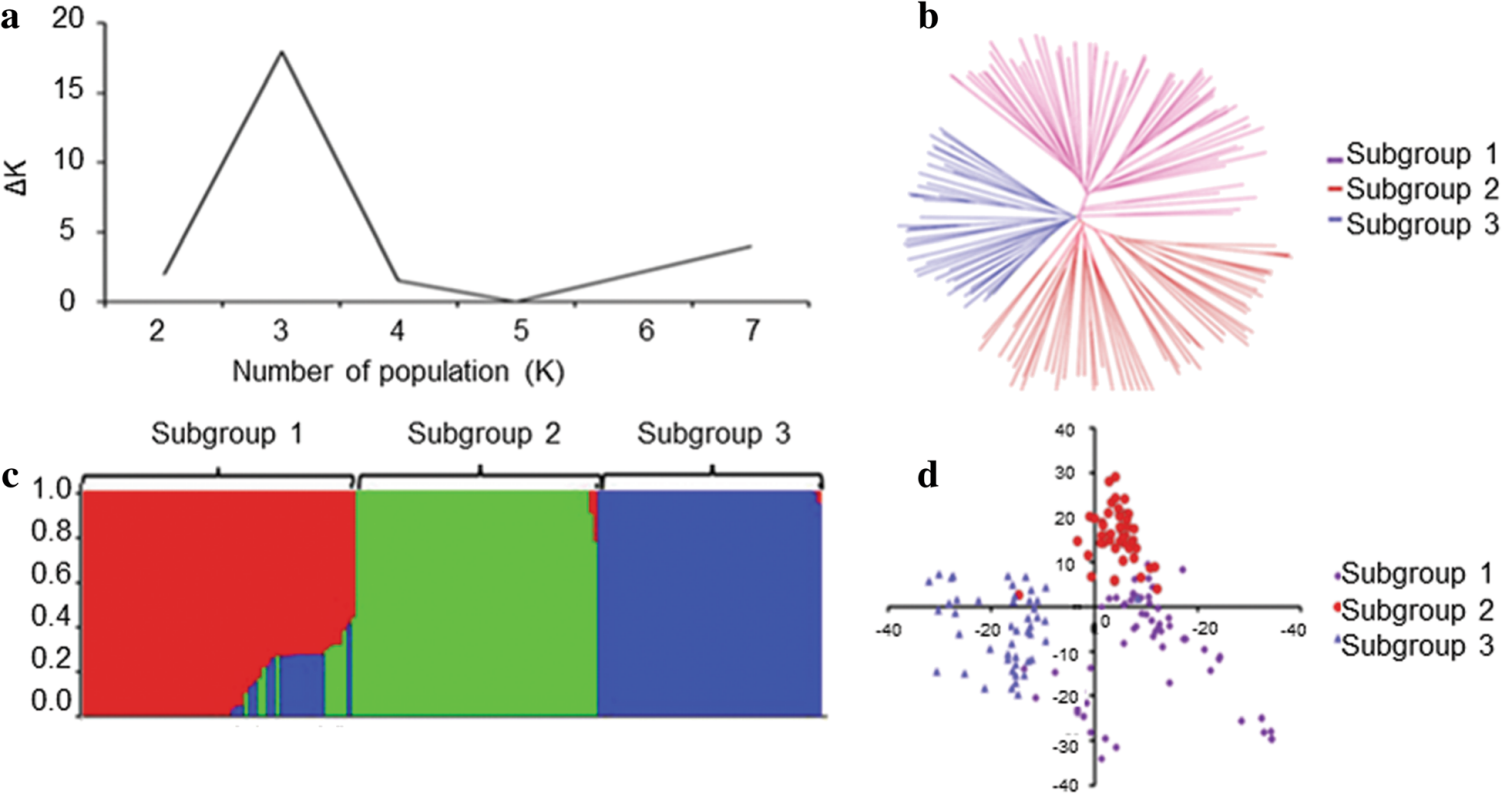
Population structure analysis of 166 wheat accessions. **a** Estimated *∆*K over five repeats of structure analysis; **b** three subgroups inferred by structure analysis; **c** neighbor-joining (NJ) tree; **d** principal components analysis (PCA) plots

In the plot of K against ΔK, a break in the slope was observed at K = 3 followed by flattening of the curve, indicating that this panel consists of three subgroups, which was consistent with the results of principal components analysis (PCA) and neighbor-joining (NJ) tree analysis (Fig. 1). Subgroup I, the largest group with 62 accessions, was dominated by Shandong and foreign cultivars; Subgroup II consisted of 54 accessions, mainly comprising varieties from Henan, Anhui and Shaanxi provinces; Subgroup III had 50 accessions, most of which were from Henan province (Additional file 1: Table S1).

In total, 12,324 markers from the 90 K and 660 K SNP arrays were used to evaluate LD decay for the whole genome as well as the A, B and D genomes separately. Around 14.3% of all pairs of loci were in significant LD (*P* < 0.001) with average *r* ^2^ of 0.174 on a genome-wide level by the 90 K and 660 K SNP assays. The B genome contained the highest percentage of significant markers (44.2%), followed by the A (33.6%) and D (22.2%) genomes. The scatter plots of *r* ^2^ against physical distance (Mb) indicated a clear LD decay with increasing physical distance (Additional file 3: Figure S1). According to [28], the critical value for significance of *r* ^2^ was evaluated at 0.079, 0.083, 0.095 and 0.082 for the A, B, D and whole genomes, respectively. The point at which the LOESS curve intercepts the critical *r* ^2^ was determined as the average LD decay of the panel [28]. Based on this criterion, LD decay distance was about 8 Mb for the whole genome. The highest LD decay was observed in the D genome (11 Mb), followed by the A (6 Mb) and B (4 Mb) genomes (Additional file 3: Figure S1).

### Phenotypic variations for black point reaction in the field

Continuous variation was observed across five environments (Additional file 4: Figure S2; Additional file 5: Table S3). The resulting best linear unbiased predictors (BLUPs) for black point scores across all environments ranged from 1.6 to 80.6% with an average of 23.3% (Additional file 4: Figure S2; Additional file 6: Figure S3), presenting a wide range of reactions for black point and indicating that this diversity panel was ideal for conducting GWAS. Analysis of variance (ANOVA) for black point scores revealed significant differences (*P* ≤ 0.001) among genotypes (G), environments, and genotype × environment (G × E) interactions (Table 1). The broad sense heritability (*h* ^*2*^) estimate for black point scores across all five environments was 0.62, indicating that much of the phenotypic variation was derived from genetic factors and therefore suitable for further association mapping.

**Table 1:** Analysis of variance of black point scores in 166 wheat accessions

Source of variation	*df*	Mean square	*F* value
Replicates (nested in environments)	9	3896	7.4***
Environments	4	499,230	480.6***
Genotypes	165	5326	35.3***
Genotypes × Environments	660	798	4.9***
Error	1650	153	

***significant at *P* < 0.0001

### Marker-trait association (MTA) analysis

The MTAs analyzed by the mixed linear model (MLM) in Tassel v5.0 [36] and the FarmCPU [37] were shown in Additional file 7: Table S4 and Additional file 8: Table S5, respectively. Twenty-five loci (221 MTAs) identified by both the Tassel v5.0 and FarmCPU were considered to be more reliable (Table 2, Additional file 9: Table S6); these were distributed on chromosomes 2A, 2B, 3A, 3B (2), 3D, 4B (2), 5A (3), 5B (3), 6A, 6B, 6D, 7A (5), 7B and 7D (2), respectively (Table 2, Additional file 9: Table S6), explaining phenotypic variation ranging from 7.9 to 18.0%. Among these loci, 13 on chromosomes 2A, 2B, 3A, 3B (2), 3D, 4B, 5A (2), 5B, 7A, 7B and 7D were detected in two or more environments (Table 2, Additional file 9: Table S6). The maximum number of loci were found in the A genome (11), followed by the B genome (10), whereas only four loci were identified in the D genome (Table 2; Additional file 9: Table S6).

**Table 2:** Loci for black point resistance in 166 wheat accessions identified by both the Tassel v5.0 and FarmCPU

Marker^a^	Chr^b^	Physical interval^c^ (bp)	Environment ^d^	SNP^e^	*P*-value^f^	*R* ^*2*g^ (%)	QTL/gene^h^
*IWB22408*	2A	709,831,643-709,831,743	E1, E2, E3, E4, E6	T/C	1.2–9.8 E^−04^	7.9–14.7	*QBp.caas-2AL* [23]; *QPPO.caas-2AL* [55]
*PPO-A1*	2A	712,188,721–712,187,200	E1, E2, E3, E4, E6	–	2.4–5.5 E^−04^	9.9–11.6	*PPO-A1* [56]
*AX_108,951,749*	2B	714,389,068–714,388,998	E2, E3, E4, E6	T/C	2.0–7.3 E^−04^	8.8–11.5	*QBp.caas-2BL* [23]
*AX_111,053,669*	3A	9,605,904–9,605,974	E2, E3, E4, E6	A/G	2.4–8.6 E^−04^	8.3–10.4	
*AX_108826477*	3B	58,767,930–58,768,000	E1, E3	A/C	1.5–9.7 E^−04^	7.9–11.0	
*AX_108,797,097*	3B	695,967,481–695,967,411	E1, E2, E3, E6	A/G	1.2–9.6 E^−04^	8.0–11.9	*QBp.caas-3BL* [23]
*AX_110941533*	3D	4,066,092–4,066,162	E2, E3, E4, E6	A/C	4.1–9.4 E^−04^	8.2–9.7	
*AX_108983386*	4B	6,961,084–6,961,154	E5	C/G	1.7–9.4 E^−05^	8.0–10.5	
*AX_111488843*	4B	504,944,902–504,944,832	E1, E2, E3, E6	A/T	7.0 E^−06^-2.6 E^−04^	9.9–15.5	
*IWB8709*	5A	32,887,598–32,887,698	E3	A/G	1.9–5.3 E^−04^	8–8.9	*QBp.caas-5AS* [23]; *QPod.caas-5AS* [57]
*AX_109316564*	5A	535,780,381–535,780,311	E1, E3, E6	T/G	4.8–9.9 E^−04^	8.1–11.4	
*IWA2223*	5A	592,276,555–592,276,708	E1, E2, E3, E4, E6	A/G	3.4 E^−06^-9.4 E^−04^	8.0–18.0	
*IWA5214*	5B	302,177,272–302,177,428	E2	A/C	8.60 E^−04^	8.1	
*AX_110617778*	5B	531,539,253–531,539,323	E2, E3	A/T	1.4–9.5 E^−04^	8.0–11.5	
*AX_110056162*	5B	556,183,885–556,183,955	E5	T/C	4.2–7.3 E^−04^	8.3–11.0	
*AX_108,821,301*	6A	94,2114,60–94,211,390	E4	C/G	1.4–8.0 E^−04^	9.1–11.2	*QBp.caas-6A* [23]
*AX_110578177*	6B	676,210,414–676,210,344	E1	A/T	4.2–5.1 E^−04^	8.6–8.9	
*AX_109359792*	6D	217,194,463–217,194,533	E4	A/G	6.30 E^−04^	8.5	
*AX_111086566*	7A	88,862,791–88,862,721	E1, E6	T/C	2.2–8.8 E^−04^	8.6–11.6	
*AX_108743156*	7A	136,398,412–136,398,482	E4, E5, E6	A/G	5.0–9.8 E^−04^	7.9–9.2	
*AX_109,311,326*	7A	609,508,901–609,508,971	E2	T/C	8.50 E^−04^	8.2	*QBp.caas-7AL.2* [23]
*AX_111042346*	7A	670,876,731–670,876,661	E5, E6	C/G	2.6–4.9 E^−04^	9.0–12.8	
*AX_109491960*	7A	70,8211,110–708,211,040	E4	A/C	1.7–9.1 E^−04^	8.4–11.2	
*AX_108870509*	7B	729,224,017–729,224,087	E1, E2, E3, E6	A/G	3.0 E^−05^-8.6 E^−04^	8.0–13.0	
*AX_109370330*	7D	129,917,622–129,917,692	E1, E3	A/C	5.2–9.5 E^−04^	8.7–9.6	
*AX_109033824*	7D	615,826,844–615,826,914	E5	A/C	8.30 E^−04^	8.2	

^a^Representative markers at the resistance loci

^b^Chr: Chromosome

^c^The physical positions of SNP markers based on wheat genome sequences from the International Wheat Genome Sequencing Consortium (IWGSC, http://www.wheatgenome.org/)

^d^E1: Anyang 2013; E2: Anyang 2014; E3: Anyang 2015; E4: Suixi 2013; E5: Suixi 2014; E6: Best linear unbiased prediction (BLUP) calculated from all five environments. The data from the results of Tassel v5.0

^e^Favorable allele (SNP) is underlined

^f^The *P*-values were calculated by the Tassel v5.0

^g^Percentage of phenotypic variance explained by the MTA from the results of Tassel v5.0

^h^The previously reported QTL or genes within the same chromosomal regions

**Fig. 2:**
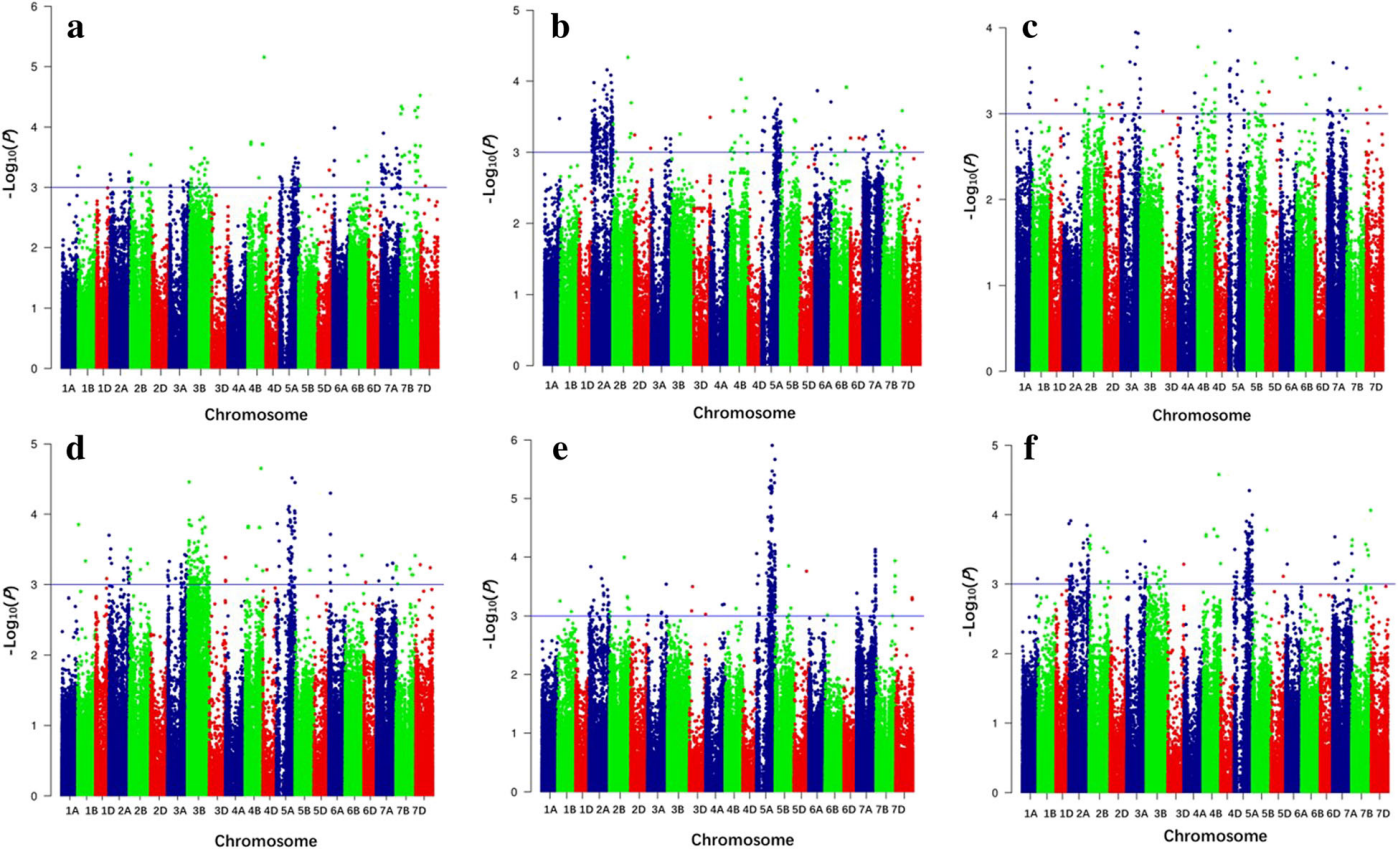
Manhattan plots for black point resistance in 166 wheat accessions by the mixed linear model (MLM) in Tassel v5.0. **a** Anyang 2013; **b** Anyang 2014; **c** Anyang 2015; **d** Suixi 2013; **e** Suixi 2014; **f** Best linear unbiased prediction (BLUP) values for black point scores calculated from all five environments. The -log_10_ (*P*) values from a genome-wide scan are plotted against positions on each of the 21 chromosomes. Horizontal lines indicate genome-wide significance thresholds

**Fig. 3:**
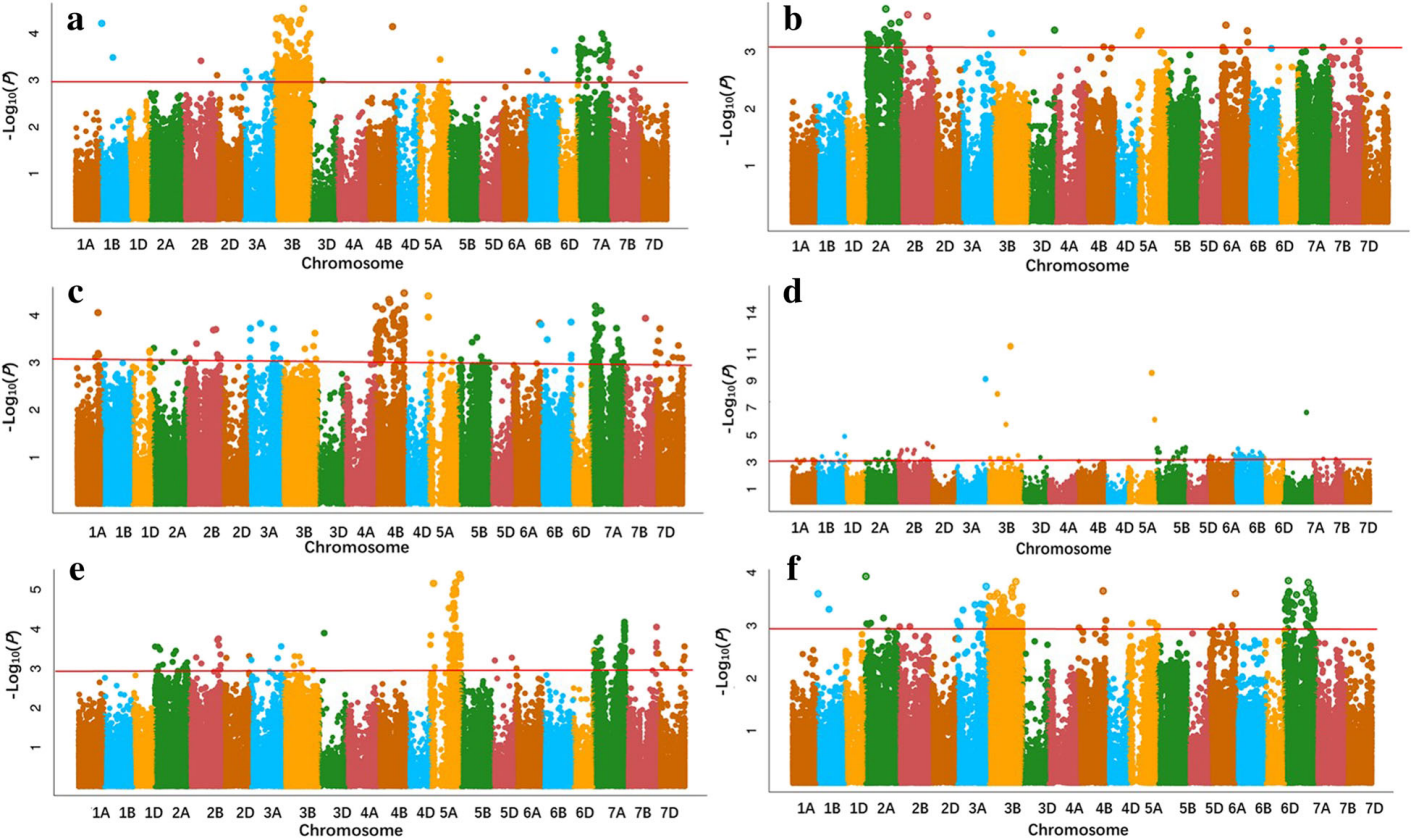
Manhattan plots for black point resistance in 166 wheat accessions by the FarmCPU. **a** Anyang 2013; **b** Anyang 2014; **c** Anyang 2015; **d** Suixi 2013; **e** Suixi 2014; **f** Best linear unbiased prediction (BLUP) values for black point scores calculated from all five environments. The -log_10_ (*P*) values from a genome-wide scan are plotted against positions on each of the 21 chromosomes. Horizontal lines indicate genome-wide significance thresholds

Kinship-adjusted Manhattan plot summarizing the analysis of black point scores by Tassel v5.0 and FarmCPU are shown in Fig. 2 and Fig. 3, respectively. The quantile-quantile (Q-Q) plot representing expected and observed probabilities of getting associations of SNPs by Tassel v5.0 and FarmCPU are presented in Additional file 10: Figure S4 and Additional file 11: Figure S5, respectively. The LD patterns along 2A, 2B, 3A, 3B, 4B, 5A, 5B, 6A, 6B, 6D, 7A, 7B and 7D can be visualized as heatmaps in Additional file 12: Figure S6.

### Relationship between black point reaction and the number of resistance alleles

**Fig. 4:**
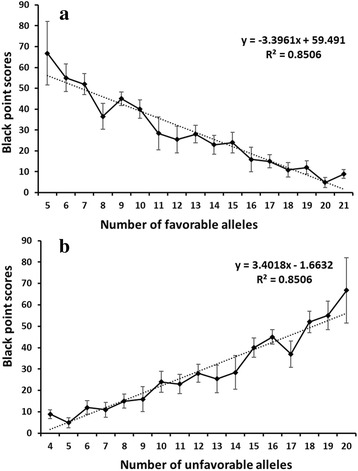
Linear regression between the number of favorable alleles (**a**) and unfavorable alleles (**b**) and the BLUP values for black point scores

To further understand the combined effects of alleles on reaction to black point, we examined the number of favorable alleles in each accession. The numbers of favorable alleles in single accessions ranged from 5 to 21, compared to 4 to 20 unfavorable alleles (Additional file 1: Table S1). The relationships between black point BLUP values and numbers of favorable and unfavorable alleles estimated by linear regression showed a dependence of black point BLUP values on the number of favorable alleles with *r* ^*2*^ = 0.85 (Fig. 4a), and number of unfavorable alleles with *r* ^*2*^ = 0.85 (Fig. 4b). Thus, accessions with more favorable alleles and less unfavorable alleles were more resistant to black point.

## Discussion

The diversity panel, including released cultivars, advanced lines and landraces from different ecological regions, thus had a high genetic diversity with a wide range of reaction to black point. Our data showed that 86.1% (143) of the 166 accessions were susceptible to black point (black point score > 10%), indicating that black point is a considerable threat to wheat production throughout the world. However, most of the previous studies for black point were mainly conducted on pathogen identification, biological characteristics, disease cycle and control [7, 13, 38]. Thus, it is necessary to select cultivars highly resistant to black point and to identify markers significantly associated with resistance to facilitate breeding for resistance by MAS.

### Genetic diversity, population structure and linkage disequilibrium

The mean genetic diversity and PIC of 0.356 and 0.285, respectively, indicated higher polymorphism than in previous reports [39, 40]. Our diversity panel thus has high genetic diversity and approximately reflected the genetic diversity in winter wheat from the Yellow and Huai River Valleys Facultative Wheat Region. More than 56% of SNPs had PIC of 0.20–0.40, which is deemed as a suitable range for GWAS [41]. Furthermore, the A and B genomes had higher genetic diversity and PIC than the D genome, consistent with previous reports [30, 40] (Additional file 2: Table S2). All results indicated that our diversity panel has high genetic diversity and was suitable for GWAS.

The diversity panel could be divided into three subgroups (Fig. 1), and the characterization of the subgroups was largely consistent with geographic origins and pedigrees. For example, Zhongmai 871, Zhongmai 875 and Zhongmai 895, which were derived from Zhoumai 16, clustered with Zhoumai 16 in group 3 (Additional file 1: Table S1). Numerous studies have shown that the lack of appropriate correction for population structure can lead to spurious MTAs [42–45]. Consequently, to eliminate spurious MTAs resulting from population structure, subpopulation data (Q matrix) were considered as fixed-effect factors, whereas the kinship matrix was considered as a random-effect factor, and a MLM implemented in Tassel v5.0 and FarmCPU were adopted for association analysis in the current study [36].

The LD decay affects the precision of GWAS and this is influenced by many factors like population structure, allele frequency, recombination rate and selection [44, 46, 47]. Previous studies reported that LD decay in common wheat ranged between 1.5–15 cM using SSR [28, 46, 48], DArT [33] or SNP [30, 47] markers. In this panel, the LD decay was about 8 Mb for the whole genome (Additional file 3: Figure S1), consistent with previous reports. The LD decay of the D genome (11 Mb) was higher than the A (6 Mb) and B (4 Mb) genomes (Additional file 3: Figure S1), also consistent with previous studies [47–49], suggesting that fewer markers are needed for GWAS in the D genome than the A and B genomes. The marker densities for the A, B and D genomes were 0.099, 0.042 and 0.202 Mb/marker, and thus highly reliable for detecting MTAs with respect to LD decay in the diversity panel according to Breseghello and Sorrells [28]. The reason for the high LD of the D genome is mainly due to limited infusion of *Aegilops tauschii* in the evolutionary history of common wheat [38, 49]. The average *r* ^2^ (0.174) values observed between linked loci pairs were higher than in previous studies [46, 50]. Reif et al. [51] reported that LD (*r* ^2^) is expected to be higher in released cultivars than landraces. Moreover, Würschum et al. [52] indicated that QTL with small effect can be detected at higher LD (*r* ^2^), whereas only QTL with large effects can be detected at lower LD (*r* ^2^). Our results thus suggested a high mapping resolution and strong QTL detection power for black point resistance.

### Comparison of the 90 K and 660 K SNP arrays

One of the key factors for GWAS is high marker density in whole genomes because sparse coverage reduces the power of marker identification [53]. Although the 90 K SNP array has emerged as a promising choice for high-density, low cost genotyping [34, 54], the presence of large gaps, particularly low coverage for the D genome, reduces the power of marker identification and decreases the precision of QTL mapping. To resolve the problem, the GWAS for black point resistance was performed using 259,922 markers from the 90 K and 660 K SNP arrays, providing a greater coverage of the genome. Only 8 loci were identified by the SNPs from 90 K array, whereas 23 were detected by the 660 K SNP, indicating that the 660 K SNP array with its much higher marker density had a significant advantage in GWAS.

### Marker-trait associations

Some black point resistance QTL were previously identified by bi-parental linkage mapping [22, 23], allowing for a comparison between loci identified in the present study and known QTL. Liu et al. [23] found seven stable black point resistance QTL on chromosomes 2AL, 2BL, 3BL, 5AS, 6A and 7AL (2) in a Linmai 2/Zhong 892 RIL population, which overlapped with loci identified in our study on chromosomes 2AL (*IWB22408*, 709.8 Mb), 2B (*AX-108951749*, 714.3 Mb), 3BL (*AX-108797097*, 695.9 Mb), 5AS (*IWB8709*, 32.8 Mb), 6A (*AX-108821301*, 94.2 Mb), and 7A (*AX-109311326*, 609.5 Mb) (Tables 2, Additional file 9: Table S6), indicating that GWAS and linkage mapping are complementary in identifying genes. Lehmensiek et al. [22] detected eight black point resistance QTL explaining 4 to 18% of the phenotypic variation on chromosomes 1D, 2A, 2B, 2D, 3D, 4A, 5A and 7A in Sunco/Tasman and Cascades/AUS1408 doubled haploid (DH) populations by SSR markers. We also identified 11 unique loci in 2A, 2B, 3D, 5A (3) and 7A (5). The loci on chromosomes 2AL (*IWB22408*, bin C-2AL1–0.85) and 2BL (*AX-108951749*, bin 2BL6–0.89-1.00) coincided with the QTL detected by Lehmensiek et al. [22] in chromosomes 2A (*Xgwm312*, bin C-2AL1–0.85) and 2B (*Xgwm319*, bin 2BL6–0.89-1.00) (Table 2, Additional file 9: Table S6). However, not all of the QTL detected in linkage analysis were found in GWAS, such as *QBp.caas-3AL* and *QBp.caa*s-7BS [23]. The reasons for this could be that (a) some QTL may have segregated at low frequency, or not at all in our association panel, and (b) results from the different marker platforms are difficult to align in the absence of complete genome sequences of diverse wheat cultivars.

Oxidases, such as PPO [15] and POD [17], could have enhanced the development of black point. The PPO gene (*Ppo-A1*) mapped to the long arm of chromosome 2AL in the interval *IWB59334*-*IWB5777* (706.2–715.3 Mb) [55], overlapped with the loci on chromosome 2AL (*IWB22408*, 709.8 Mb) in our study. In addition, the *Ppo-A1*-specific marker *PPO18* [56] was also significantly associated with black point resistance (Table 2). Furthermore, the SNP marker *IWA5214* (302.2 Mb) on chromosome 5BL was significantly associated with both black point resistance and PPO activity (Zhai et al. unpublished data). Wei et al. [57] identified a QTL for POD activity on chromosome 5AS (15.9–36.9 Mb) using a RIL population derived from Doumai/Shi 4185, corresponding to the major loci detected on chromosome 5AS (*IWB8705*, 32.8 Mb) in this study (Additional file 9: Table S6). Shi et al. (unpublished data) identified a locus for POD activity on chromosome 2AL (*IWB59334*, 715.3 Mb) by GWAS, which overlapped with the locus on chromosome 2AL (*IWB22408*, 709.8 Mb). Thus, the GWAS results confirmed previous reports implicating phenol metabolism enzymes like PPO and POD in development of black point [15, 17, 18].

As the genetics of black point reaction are still poorly understood, the remaining 18 loci identified on chromosomes 3A, 3B, 3D, 4B (2), 5A (2), 5B (2), 6B, 6D, 7A (4), 7B and 7D (2) represent potentially new resistance QTL (Table 2); these may contribute to better understand of the architecture of black point reaction and provide more opportunities for resistance breeding. The above results demonstrated that GWAS was a powerful and reliable tool for identification of black point resistance genes.

### Candidate genes for black point resistance

To identify candidate genes for black point resistance, the flanking sequences of SNP markers significantly associated with black point reaction were imported to Blast2Go software, and used as queries to BLAST against the National Center for Biotechnology Information (NCBI) and European Nucleotide Archive (ENA) databases; six candidate genes were identified (Table 3). Bioinformatics analysis indicated that SNP marker *AX-111518195* on chromosome 2AL corresponded to peroxisomal biogenesis factor 2, an important gene for biosynthesis of peroxidase, which can accelerate oxidation of phenolic compounds to quinones and is crucial for phenolic metabolism and melanin synthesis [18, 58]. In addition, the gene-specific marker *PPO18* for *Ppo-A1* [56] overlapping with the SNP loci on chromosome 2AL was also significantly associated with black point reaction. Fuerst et al. [18] reported that PPO catalyzes oxidation of phenolic compounds to melanins and quinines that may contribute to black point development. Thus, *Ppo-A1* is a candidate gene for this locus. Marker *AX-95684401* on chromosome 5A corresponded to a gibberellin (GA) biosynthetic process protein. GA plays an important role in modulating disease reaction throughout plant development and affects black point development by influencing seed germination [59]. Marker *IWA5463* on chromosome 2AL corresponds to an F-box repeat protein, which may affect black point development by regulating signal transduction of gibberellin [59, 60]. F-box proteins have also been implicated in response to various pathogens through targeting substrates in the degradation machinery [61]. Two SNP markers (*AX-108951749* on 2B and *IWA2223* on 5AL) encode serine/threonine-protein kinases, which trigger multiple physiological and biochemical reactions in response to abiotic and biotic stresses by mediating perception and transduction of external environmental signals [62, 63]. We also identified a candidate gene encoding a disease resistance RPP8-like protein (*AX-111053669* on chromosome 3A), which had been proposed to play an essential role in regulation of responses to a variety of external stimuli, including stress [64]. Bioinformatics analysis of trait-associated SNPs was proven to be an effective tool to find candidate genes for complex agronomic traits [34]. However, black point is a consequence of complicated biological processes and the mechanism of black point formation remains unclear; more detailed experimental analyses are needed to confirm the roles of candidate genes in black point resistance.

**Table 3:** Candidate genes for SNPs significantly associated with black point resistance

Chromosome	Marker	Candidate gene	Sequence similarity (%)	Sequence coverage (%)	Quality parameters
2AL	*IWA5463*	F-box repeat	98	98	4 E^−36^
2AL	*PPO-18*	Polyphenol oxidase (*PPO-A1*)	–	–	–
2AL	*AX-111518195*	Peroxisomal biogenesis factor 2	97	97	4 E^−12^
2B	*AX-108951749*	Serine/threonine-protein kinase	97	96	6 E^−06^
3A	*AX-111053669*	Disease resistance RPP8-like protein	97	96	1 E^−08^
5AL	*IWA2223*	Serine/threonine-protein kinase	100	99	8 E^−39^
5A	*AX-95684401*	Gibberellin biosynthetic process	97	100	4 E^−07^

### Application of MTAs for black point resistance in wheat breeding

It is difficult to select highly resistant lines at the early stages of a breeding program in the field due to the fact that black point symptoms can be assessed only on mature seed after harvest and are highly affected by environment. A significant additive effect was identified from the linear regression between black point resistance and the number of favorable alleles, indicating that pyramiding of favorable alleles will enhance resistance. Markers significantly associated with complex traits identified by GWAS or QTL mapping can be converted into kompetitive allele-specific PCR (KASP) markers for SNP validation, MAS and QTL fine mapping [65, 66]. Semi-thermal asymmetric reverse PCR (STARP) also provides a new scalable, flexible and cost-effective approach for using SNP markers in MAS [67]. QTL with consistent effects across multiple environments should be useful for MAS [68]. Thirteen of the 25 loci identified in this study were detected in two or more environments and should be suitable for MAS. Some accessions with higher black point resistance and relatively high number of resistance alleles and excellent agronomic traits, such as Kitanokaori, Norin 67, Yumai 21, Yannong 19, Zhoumai 19, and Zhongmai 871 (Additional file 13: Table S7), should be good parental lines for breeding. Our follow-up studies will focus on validating the effects of these QTL and developing friendly, tightly linked markers that can be used in resistance breeding.

## Conclusions

In the present study, a GWAS for black point resistance in a diversity panel was conducted with the 90 K and 660 K SNP arrays. Twenty-five resistance loci explained 7.9–18.0% of the phenotypic variations, demonstrating that GWAS can be used as a powerful and reliable tool for dissecting genes in wheat. The markers significantly associated with black point resistance and the accessions with a higher number of resistance alleles can be used as valuable markers and excellent parent material for resistance breeding. This study improves our understanding of the genetic architecture of black point resistance in common wheat.

## Methods

### Plant materials and field trials

The association panel used in the present study contained 166 diverse cultivars, comprising 144 accessions from the Yellow and Huai River Valley Facultative Wheat Region of China, and 22 accessions from five other countries, including Italy (9), Argentina (7), Japan (4), Australia (1) and Turkey (1) (Additional file 1: Table S1). All accessions were grown at Anyang (35°12′N, 113°37′E) in Henan province during the 2012–2013 and 2013–2014 cropping seasons, and Suixi (33°17′N, 116°23′E) in Anhui province during 2012–2013, 2013–2014 and 2014–2015. Field trials were conducted in randomized complete blocks with three replicates at all locations. Each plot contained three 2 m rows spaced 20 cm apart. Agronomic management followed local practices. All wheat accessions are deposited in the National Genebank of China, Chinese Academy of Agricultural Sciences, and available after approval.

### Phenotypic evaluation and statistical analysis

After harvest and threshing three samples of 200 grains were selected from each of the three replicates of each accession, and the percentages of kernels with black point symptoms were determined and averaged. BLUPs across five environments were used as the phenotypic values for association mapping to eliminate environmental effects. BLUP estimation was calculated using the MIXED procedure (PROCMIXED) in SAS v9.3 (SAS Institute, http://www.sas.com).

ANOVA was performed using SAS v9.3 (SAS Institute, http://www.sas.com). Variance components were used to calculate broad sense heritability (*h* ^*2*^) of black point scores as *h* ^*2*^ = *σ*_*g*_^*2*^/ (*σ*_*g*_^*2*^ + *σ*_*ge*_^*2*^/*r* + *σ*_*ε*_^*2*^/*re*), where *σ*_*g*_^*2*^, *σ*_*ge*_^*2*^ and *σ*_*ε*_^*2*^ represent the genotype, genotype × environment interaction and residual error variances, respectively, and *e* and *r* were the numbers of environments and replicates per environment, respectively.

### Genotyping and quality control

Total genomic DNA for SNP arrays was extracted from five bulked young leaves from each accession using a modified CTAB procedure [69]. The 166 accessions were genotyped using both the Illumina wheat 90 K SNP (containing 81,587 SNPs) and Affymetrix 660 K SNP (containing 630,517 SNPs) arrays by Capital Bio Corporation, Beijing, China (http://www.capitalbiotech.com/). Accuracy of SNP clustering was validated visually. MAF, genetic diversity and PIC were computed by PowerMarker v3.25 [70] (http://statgen.ncsu.edu/powermarker/). To avoid spurious MTAs, SNP markers with MAF < 0.05 and missing data >20% were excluded from further analyses. The physical positions of SNP markers from the wheat 90 K and 660 K SNP arrays were obtained from the International Wheat Genome Sequencing Consortium website (IWGSC, http://www.wheatgenome.org/), and markers from two SNP arrays were integrated into a common physical map for GWAS.

### Population structure

Population structure was analyzed using 2000 polymorphic SNP markers from the 90 K and 660 K SNP arrays with Structure v2.3.4 [41] (http://pritchardlab.stanford.edu/structure.html), which implements a model-based Bayesian cluster analysis. Five independent runs for each K value from 2 to 12 were performed based on an admixture model. Each run was carried out with 100,000 recorded Markov-Chain iterations and 10,000 burn-in periods. An adhoc quantity statistic ΔK based on the rate of change in log probability of data between successive K values [71] was used to predict the real number of subpopulations. PCA and NJ trees were also used to validate population stratification with the software Tassel v5.0 [44] and PowerMarker v3.25 [70] (http://www.maizegenetics.net).

### Linkage disequilibrium

LD among markers was calculated using the full matrix and sliding window options in Tassel v5.0 with 12,324 evenly distributed SNP markers. The positions of these markers were based on the physical map mentioned above. Pairwise LD was measured using squared allele-frequency correlations *r* ^2^, and significance of pair-wise LD (*P*-values) was measured by Tassel v5.0 with 1000 permutations. The *r* ^*2*^ values were plotted against physical distance and a LOESS curve was fitted to the plot to show the association between LD decay and physical map distance. The critical value of *r* ^2^ beyond which the LD was likely to be caused by genetic linkage was determined by taking the 95th percentile in the distribution of *r* ^2^ of the selected loci [28]. The intersection of the fitted curve of *r* ^2^ values with this threshold was considered as the estimate of LD range.

### Genome-wide association analysis

Associations between genotypic and phenotypic data were analyzed using the kinship matrix in a MLM by Tassel v5.0 to control background variation and eliminate the spurious MTAs. In MLM analysis, the kinship matrix (K matrix) was considered a random-effect factor, whereas the subpopulation data (Q matrix) was considered a fixed-effect factor [43]. The K matrix was calculated by the software Tassel v5.0 and the Q matrix was inferred by the program Structure v2.3.4. The *P* value determining whether a SNP marker was associated with the trait and the *R* ^2^ indicating the variation explained by the marker was recorded. The GWAS was also analyzed using the FarmCPU software [37] by R Language (https://www.r-project.org/). Bonferroni-Holm correction [72] for multiple testing (α = 0.05) was too conserved and no significant MTAs were detected with this criterion. Therefore, markers with an adjusted -log_10_ (*P*-value) ≥ 3.0 were regarded as significant markers for black point reaction [73–75], as shown in Manhattan plots using the ggplot2 code in R Language. Important *P* value distributions (observed *P* values plotted against expected *P* values) were shown in Q-Q plots.

We checked the LD (*r* ^2^) among markers significantly associated with black point reaction on the same chromosomes to compare the resistance loci. LD block on the same chromosome were computed and visualized by Haploview v4.2 [76] (www.broadinstitute.org/haploview/haploview). To compare resistance loci identified in the present study with known genes/QTL, deletion bin information for SSR and SNP markers was obtained following [23].

### The effect of favorable alleles on black point resistance

Each locus comprises two alleles based on SNP marker a single base substitution, transition or transversion. Alleles with positive effects leading to higher black point resistance are referred to as “favorable alleles”, and those leading to lower resistance are “unfavorable alleles”. The representative SNPs at the resistance loci were used to count the frequencies of favorable and unfavorable alleles and their allelic effects were determined (Table 2). Regression analysis between favorable, unfavorable alleles and black point scores were conducted using the line chart function in Microsoft Excel 2016.

### In silico annotation of SNPs

To identify candidate genes or putative protein functions of SNP flanking-regions, the flanking sequences corresponding to the SNP markers significantly associated with black point resistance were used in BLASTn and BLASTx searches against ENA (http://www.ebi.ac.uk/ena) and NCBI (http://www.ncbi.nlm.nih.gov/) databases. Sequences were imported to Blast2Go software (https://www.blast2go.com/) in fasta formats that were blasted, mapped and annotated using standard parameters embedded in the software.

## Additional files

Additional file 1: Table S1. The 166 wheat accessions used in the genome-wide association study (GWAS) for black point reaction and their origins. (DOCX 27 kb)

Additional file 2: Table S2. Basic statistical analysis of SNP markers used for genome-wide association study (GWAS) of 166 wheat accessions. (DOCX 19 kb)

Additional file 3: Figure S1. LD decay estimated in 166 wheat accessions based on 12,324 markers from the 90 K and 660 K SNP arrays. (DOCX 97 kb)

Additional file 4: Figure S2. Black point scores evaluated for 166 wheat accessions. Reported values are the best linear unbiased predictions (BLUP) value for black point scores across five environments. (DOCX 430 kb)

Additional file 5: Table S3. The black point scores of the 166 wheat accessions across the five environments. (XLSX 25 kb)

Additional file 6: Figure S3. Frequency distributions for black point scores for 166 wheat accessions in five environments. (a) Anyang 2013; (b) Anyang 2014; (c) Anyang 2015; (d) Suixi 2013; (e) Suixi 2014; (f) Best linear unbiased predictions (BLUP) value for black point scores across five environments. (DOCX 292 kb)

Additional file 7: Table S4. Marker-trait associations (MTAs) for black point reaction in 166 wheat accessions analyzed by the mixed linear model (MLM) in Tassel v5.0. (XLSX 80 kb)

Additional file 8: Table S5. Marker-trait associations (MTAs) for black point resistance in 166 wheat accessions analyzed by the FarmCPU. (XLSX 94 kb)

Additional file 9: Table S6. Marker-trait associations (MTAs) for black point resistance in 166 wheat accessions identified by both the Tassel v5.0 and FarmCPU. (XLSX 18 kb)

Additional file 10: Figure S4. Quantile-quantile (Q-Q) plot for black point scores in 166 wheat accessions by the mixed linear model (MLM) in Tassel v5.0. (a) Anyang 2013; (b) Anyang 2014; (c) Anyang 2015; (d) Suixi 2013; (e) Suixi 2014; (f) Best linear unbiased predictions (BLUP) value for black point scores across five environments. (DOCX 682 kb)

Additional file 11: Figure S5. Quantile-quantile (Q-Q) plot for black point scores in 166 wheat accessions by the FarmCPU. (a) Anyang 2013; (b) Anyang 2014; (c) Anyang 2015; (d) Suixi 2013; (e) Suixi 2014; (f) Best linear unbiased predictions (BLUP) value for black point scores across five environments. (DOCX 379 kb)

Additional file 12: Figure S6. LD heatmap of all wheat chromosomes showing extent of pairwise linkage disequilibrium between SNP markers significantly associated with black point reactions. (DOCX 4561 kb)

Additional file 13: Table S7. Accessions with high black point resistance. (DOCX 17 kb)

## Abbreviations

ANOVA: Analysis of variance
BLUPs: Resulting best linear unbiased predictors
DH: Doubled haploid
GA: Gibberellin
GWAS: Genome-wide association study
KASP: Kompetitive allele-specific PCR
LD: Linkage disequilibrium
LOX: Lipoxygenase
MAF: Minor allele frequency
MAS: Marker-assisted selection
MLM: Mixed linear model
MTA: Marker-trait association
NJ: Neighbor-joining
PAL: Phenylalanine ammonia-lyase
PCA: Principal components analysis
PIC: Polymorphism information content
POD: Peroxidase
PPO: Polyphenol oxidase
Q-Q: Quantile-quantile
QTL: Quantitative trait loci (locus)
RIL: Recombinant inbred line
SNP: Single nucleotide polymorphism
SSR: Simple sequence repeat
STARP: Semi-thermal asymmetric reverse PCR
