# Supplementary material for: Genome-wide association mapping of black point reaction in common wheat (Triticum aestivum L.)
Source: BMC Plant Biol. 2017 Nov 23;17:220. doi: 10.1186/s12870-017-1167-3 (PMC5701291; doi:10.1186/s12870-017-1167-3)
Supplement: Supplementary file 1 — The 166 wheat accessions used in the genome-wide association study (GWAS) for black point reaction and their origins. (DOCX 27 kb) [file 12870_2017_1167_MOESM1_ESM.docx]

**Additional file 1: Table S1** The 166 wheat accessions used in the genome-wide association study (GWAS) for black point reaction and their origins

| **No.** | **Name** | **Origin** | **No. of favorable**  **alleles** | **BLUP ^a^** | **Subgroup ^b^** |
| --- | --- | --- | --- | --- | --- |
| 1 | An 1331 | Anhui | 19 | 12.5 | 2 |
| 2 | Fu 936 | Anhui | 21 | 7.6 | 2 |
| 3 | Huaimai 18 | Anhui | 18 | 12.0 | 1 |
| 4 | Huaimai 20 | Anhui | 17 | 15.7 | 1 |
| 5 | Huaimai 21 | Anhui | 17 | 12.3 | 3 |
| 6 | Su 0663 | Anhui | 13 | 25.8 | 2 |
| 7 | Sunong 6 | Anhui | 19 | 8.2 | 2 |
| 8 | Wan 23094 | Anhui | 14 | 26.8 | 2 |
| 9 | Wanmai 19 | Anhui | 18 | 12.5 | 2 |
| 10 | Wanmai 29 | Anhui | 19 | 10.2 | 2 |
| 11 | Wanmai 33 | Anhui | 12 | 27.6 | 2 |
| 12 | Wanmai 38 | Anhui | 17 | 16.5 | 1 |
| 13 | Wanmai 50 | Anhui | 9 | 36.1 | 2 |
| 14 | Wanmai 52 | Anhui | 8 | 33.8 | 2 |
| 15 | Wanmai 53 | Anhui | 9 | 37.7 | 2 |
| 16 | Gaocheng 8901 | Hebei | 7 | 40.1 | 1 |
| 17 | Gaoyou 503 | Hebei | 13 | 26.8 | 2 |
| 18 | Han 6172 | Hebei | 12 | 30.9 | 3 |
| 19 | Heng 7228 | Hebei | 18 | 13.8 | 3 |
| 20 | Hengguan 33 | Hebei | 18 | 16.9 | 3 |
| 21 | Hengguan 35 | Hebei | 16 | 26.3 | 3 |
| 22 | Jinhe 9123 | Hebei | 16 | 25.6 | 3 |
| 23 | Jishi 02-1 | Hebei | 19 | 9.4 | 2 |
| 24 | Shi 4185 | Hebei | 15 | 21.0 | 3 |
| 25 | Shijiazhuang 15 | Hebei | 12 | 11.9 | 3 |
| 26 | Shijiazhuang 8 | Hebei | 11 | 29.8 | 3 |
| 27 | Shixin 733 | Hebei | 7 | 41.4 | 2 |
| 28 | Shixin 828 | Hebei | 6 | 51.4 | 2 |
| 29 | Shiyou 17 | Hebei | 15 | 20.1 | 1 |
| 30 | 11CA40 | Henan | 13 | 29.8 | 1 |
| 31 | 85Zhong 33 | Henan | 12 | 35.4 | 3 |
| 32 | Aikang 58 | Henan | 13 | 28.5 | 3 |
| 33 | Bainong 3217 | Henan | 16 | 19.8 | 2 |
| 34 | Bainong 64 | Henan | 15 | 16.5 | 2 |
| 35 | Huapei 5 | Henan | 19 | 11.0 | 3 |
| 36 | Lankao 2 | Henan | 15 | 26.7 | 3 |
| 37 | Lankao 24 | Henan | 15 | 18.5 | 3 |
| 38 | Lankao 906 | Henan | 14 | 25.2 | 3 |
| 39 | Luohan 2 | Henan | 15 | 21.0 | 2 |
| 40 | Luomai 21 | Henan | 15 | 22.6 | 3 |
| 41 | Neixiang 188 | Henan | 19 | 11.7 | 2 |
| 42 | Neixiang 5 | Henan | 9 | 34.7 | 3 |
| 43 | St1472/506 | Henan | 12 | 23.6 | 2 |
| 44 | Xinmai 19 | Henan | 6 | 51.1 | 2 |
| 45 | Xinmai 9 | Henan | 7 | 46.4 | 2 |
| 46 | Xinmai 9408 | Henan | 9 | 39.9 | 2 |
| 47 | Yanzhan 4110 | Henan | 10 | 33.2 | 2 |
| 48 | Yumai 13 | Henan | 19 | 11.5 | 1 |
| 49 | Yumai 18 | Henan | 7 | 49.3 | 2 |
| 50 | Yumai 2 | Henan | 13 | 27.3 | 3 |
| 51 | Yumai 21 | Henan | 21 | 6.1 | 1 |
| 52 | Yumai 34 | Henan | 13 | 27.1 | 1 |
| 53 | Yumai 35 | Henan | 15 | 18.0 | 3 |
| 54 | Yumai 47 | Henan | 15 | 18.8 | 2 |
| 55 | Yumai 49 | Henan | 12 | 24.3 | 2 |
| 56 | Yumai 50 | Henan | 9 | 48.1 | 3 |
| 57 | Yumai 57 | Henan | 7 | 42.0 | 2 |
| 58 | Yumai 63 | Henan | 12 | 28.9 | 2 |
| 59 | Yumai 7 | Henan | 14 | 21.0 | 3 |
| 60 | Zheng 9023 | Henan | 14 | 26.5 | 2 |
| 61 | Zhengmai 366 | Henan | 21 | 11.3 | 2 |
| 62 | Zhengzhou 3 | Henan | 14 | 20.5 | 2 |
| 63 | Zhong 892 | Henan | 13 | 34.2 | 3 |
| 64 | Zhongmai 871 | Henan | 19 | 6.8 | 3 |
| 65 | Zhongmai 875 | Henan | 15 | 27.9 | 3 |
| 66 | Zhongmai 895 | Henan | 15 | 28.9 | 3 |
| 67 | Zhongyu 5 | Henan | 18 | 9.7 | 3 |
| 68 | Zhongyu 9 | Henan | 16 | 20.4 | 3 |
| 69 | Zhou 8425B | Henan | 13 | 28.1 | 3 |
| 70 | Zhoumai 11 | Henan | 5 | 67.8 | 3 |
| 71 | Zhoumai 12 | Henan | 11 | 35.3 | 3 |
| 72 | Zhoumai 13 | Henan | 17 | 14.4 | 3 |
| 73 | Zhoumai 16 | Henan | 11 | 36.6 | 3 |
| 74 | Zhoumai 18 | Henan | 16 | 22.1 | 3 |
| 75 | Zhoumai 19 | Henan | 17 | 11.0 | 2 |
| 76 | Zhoumai 22 | Henan | 13 | 22.1 | 3 |
| 77 | Zhoumai 23 | Henan | 14 | 26.8 | 2 |
| 78 | Zhoumai 25 | Henan | 18 | 12.7 | 3 |
| 79 | Zhoumai 26 | Henan | 17 | 16.0 | 3 |
| 80 | Zhoumai 28 | Henan | 12 | 29.7 | 3 |
| 81 | Zhoumai 30 | Henan | 9 | 44.3 | 3 |
| 82 | Zhoumai 31 | Henan | 10 | 45.9 | 2 |
| 83 | Zhoumai 32 | Henan | 12 | 37.3 | 3 |
| 84 | Aifeng 3 | Shaanxi | 15 | 19.8 | 1 |
| 85 | Bima 1 | Shaanxi | 14 | 16.4 | 1 |
| 86 | Bima 4 | Shaanxi | 16 | 15.3 | 1 |
| 87 | Fengchan 3 | Shaanxi | 12 | 25.0 | 2 |
| 88 | Shan 150 | Shaanxi | 11 | 28.7 | 2 |
| 89 | Shan 229 | Shaanxi | 9 | 37.8 | 2 |
| 90 | Shan 253 | Shaanxi | 13 | 27.4 | 2 |
| 91 | Shan 354 | Shaanxi | 17 | 9.0 | 3 |
| 92 | Shan 512 | Shaanxi | 12 | 23.6 | 2 |
| 93 | Shan 715 | Shaanxi | 20 | 8.0 | 3 |
| 94 | Shanmai 509 | Shaanxi | 15 | 14.0 | 3 |
| 95 | Shanmai 94 | Shaanxi | 14 | 17.5 | 2 |
| 96 | Shannong 7859 | Shaanxi | 18 | 11.0 | 3 |
| 97 | Shannong 981 | Shaanxi | 14 | 23.5 | 2 |
| 98 | Shanyou 225 | Shaanxi | 16 | 14.2 | 2 |
| 99 | Wunong 148 | Shaanxi | 15 | 15.8 | 2 |
| 100 | Xiaoyan 22 | Shaanxi | 15 | 10.9 | 3 |
| 101 | Xiaoyan 54 | Shaanxi | 16 | 11.3 | 2 |
| 102 | Xiaoyan 6 | Shaanxi | 17 | 13.7 | 2 |
| 103 | Xiaoyan 81 | Shaanxi | 15 | 13.0 | 2 |
| 104 | Xinong 1376 | Shaanxi | 16 | 11.4 | 3 |
| 105 | Xinong 2000-7 | Shaanxi | 16 | 11.3 | 2 |
| 106 | Xinong 291 | Shaanxi | 16 | 7.9 | 1 |
| 107 | Xinong 88 | Shaanxi | 10 | 42.1 | 2 |
| 108 | Xinong 979-005 | Shaanxi | 20 | 6.3 | 2 |
| 109 | Jimai 19 | Shandong | 10 | 37.5 | 1 |
| 110 | Jimai 20 | Shandong | 13 | 21.3 | 1 |
| 111 | Jimai 21 | Shandong | 11 | 33.0 | 1 |
| 112 | Jimai 22 | Shandong | 15 | 19.0 | 1 |
| 113 | Jinan 13 | Shandong | 14 | 20.8 | 1 |
| 114 | Jinan 17 | Shandong | 16 | 11.8 | 1 |
| 115 | Jining 16 | Shandong | 15 | 23.7 | 1 |
| 116 | Liangxing 66 | Shandong | 16 | 17.6 | 1 |
| 117 | Liangxing 99 | Shandong | 14 | 20.9 | 1 |
| 118 | Linmai 2 | Shandong | 8 | 44.3 | 1 |
| 119 | Linmai 4 | Shandong | 13 | 27.4 | 1 |
| 120 | Luami 15 | Shandong | 12 | 21.1 | 3 |
| 121 | Lumai 11 | Shandong | 15 | 17.4 | 1 |
| 122 | Lumai 14 | Shandong | 14 | 16.3 | 1 |
| 123 | Lumai 21 | Shandong | 10 | 34.4 | 1 |
| 124 | Lumai 23 | Shandong | 13 | 25.9 | 1 |
| 125 | Lumai 5 | Shandong | 9 | 41.8 | 1 |
| 126 | Lumai 6 | Shandong | 8 | 36.2 | 2 |
| 127 | Lumai 7 | Shandong | 12 | 26.3 | 3 |
| 128 | Lumai 8 | Shandong | 10 | 36.3 | 1 |
| 129 | Lumai 9 | Shandong | 15 | 24.5 | 1 |
| 130 | Luyuan 502 | Shandong | 15 | 15.4 | 1 |
| 131 | PH 82-2 | Shandong | 19 | 9.2 | 2 |
| 132 | Shannong 20 | Shandong | 13 | 29.7 | 1 |
| 133 | Taishan 1 | Shandong | 5 | 81.5 | 1 |
| 134 | Taishan 5 | Shandong | 5 | 40.2 | 1 |
| 135 | Wennong 14 | Shandong | 8 | 28.2 | 1 |
| 136 | Wennong 5 | Shandong | 12 | 23.3 | 1 |
| 137 | Yannong 15 | Shandong | 13 | 15.9 | 1 |
| 138 | Yannong 18 | Shandong | 11 | 30.6 | 3 |
| 139 | Yannong 19 | Shandong | 18 | 9.4 | 1 |
| 140 | Zimai 12 | Shandong | 12 | 21.9 | 1 |
| 141 | Zixuan 2 | Shandong | 11 | 12.9 | 1 |
| 142 | Jinmai 61 | Shanxi | 12 | 14.3 | 1 |
| 143 | Linhan 2 | Shanxi | 14 | 29.0 | 2 |
| 144 | Linkang 12 | Shanxi | 7 | 41.5 | 3 |
| 145 | Aca 601 | Argentina | 12 | 23.4 | 1 |
| 146 | Aca 801 | Argentina | 19 | 8.4 | 1 |
| 147 | Klein Flecha | Argentina | 18 | 7.8 | 1 |
| 148 | Klein Jabal1 | Argentina | 21 | 3.6 | 1 |
| 149 | Nidera Baguette 10 | Argentina | 16 | 21.4 | 1 |
| 150 | Nidera Baguette 20 | Argentina | 21 | 5.6 | 1 |
| 151 | Prointa Colibr 1 | Argentina | 17 | 9.0 | 1 |
| 152 | Sunstate | Australia | 16 | 16.8 | 1 |
| 153 | Abbondanza | Italy | 18 | 11.3 | 1 |
| 154 | Barra | Italy | 21 | 2.1 | 1 |
| 155 | Dorico | Italy | 14 | 30.9 | 1 |
| 156 | Funo | Italy | 12 | 21.0 | 2 |
| 157 | Genio | Italy | 15 | 12.7 | 1 |
| 158 | Lampo | Italy | 12 | 33.9 | 1 |
| 159 | Libero | Italy | 10 | 37.0 | 1 |
| 160 | Mantol | Italy | 14 | 22.2 | 1 |
| 161 | Sagittario | Italy | 17 | 9.9 | 1 |
| 162 | Kanto 107 | Japan | 7 | 45.8 | 1 |
| 163 | Kitanokaori | Japan | 20 | 2.6 | 3 |
| 164 | Norin 61 | Japan | 18 | 7.3 | 1 |
| 165 | Norin 67 | Japan | 18 | 2.1 | 1 |
| 166 | Hk1/6/Nvsr3/5/Bez/  Tvr/5/Cfn/Bez//Su92  /Ci13645/3Nai60 | Turkey | 7 | 35.3 | 1 |

^a^ Best linear unbiased prediction (BLUP) values for black point scores across five environments.

^b^ Accessions were grouped into subgroups 1, 2, and 3.
