# Supplementary material for: Genome-wide association mapping of black point reaction in common wheat (Triticum aestivum L.)
Source: BMC Plant Biol. 2017 Nov 23;17:220. doi: 10.1186/s12870-017-1167-3 (PMC5701291; doi:10.1186/s12870-017-1167-3)
Supplement: Supplementary file 2 — Basic statistical analysis of SNP markers used for genome-wide association study (GWAS) of 166 wheat accessions. (DOCX 19 kb) [file 12870_2017_1167_MOESM2_ESM.docx]

**Additional file 2: Table S2** Basic statistical analysis of SNP markers used for genome-wide association study (GWAS) of 166 wheat accessions

| **Chr ^a^** | **No. of markers** | **Length ^b^**  **(Mb)** | **Density (Mb/marker)** |  | **Diversity** | |  | **PIC ^c^** | |
| --- | --- | --- | --- | --- | --- | --- | --- | --- | --- |
|  |  |  |  |  | **Mean** | **Range** |  | **Mean** | **Range** |
| 1A | 18,965 | 594.0 | 0.031 |  | 0.355 | 0.095-0.500 |  | 0.284 | 0.090-0.375 |
| 1B | 17,144 | 689.4 | 0.040 |  | 0.366 | 0.095-0.500 |  | 0.293 | 0.090-0.375 |
| 1D | 4,964 | 495.4 | 0.100 |  | 0.347 | 0.095-0.500 |  | 0.279 | 0.090-0.375 |
| 2A | 2,000 | 780.7 | 0.390 |  | 0.360 | 0.095-0.500 |  | 0.288 | 0.090-0.375 |
| 2B | 23,287 | 801.2 | 0.034 |  | 0.370 | 0.095-0.500 |  | 0.297 | 0.090-0.375 |
| 2D | 4,104 | 651.6 | 0.159 |  | 0.361 | 0.096-0.500 |  | 0.289 | 0.090-0.375 |
| 3A | 12,087 | 750.8 | 0.062 |  | 0.356 | 0.095-0.500 |  | 0.281 | 0.090-0.375 |
| 3B | 38,116 | 830.3 | 0.022 |  | 0.406 | 0.095-0.500 |  | 0.313 | 0.090-0.375 |
| 3D | 2,917 | 615.4 | 0.211 |  | 0.302 | 0.095-0.498 |  | 0.251 | 0.090-0.374 |
| 4A | 11,938 | 744.5 | 0.062 |  | 0.378 | 0.095-0.500 |  | 0.299 | 0.090-0.375 |
| 4B | 10,680 | 673.4 | 0.063 |  | 0.352 | 0.095-0.500 |  | 0.283 | 0.090-0.375 |
| 4D | 1,055 | 509.6 | 0.483 |  | 0.390 | 0.095-0.500 |  | 0.313 | 0.090-0.375 |
| 5A | 11,472 | 709.7 | 0.061 |  | 0.360 | 0.096-0.500 |  | 0.289 | 0.090-0.375 |
| 5B | 28,584 | 713.0 | 0.025 |  | 0.368 | 0.095-0.500 |  | 0.293 | 0.090-0.375 |
| 5D | 3,212 | 566.0 | 0.176 |  | 0.320 | 0.095-0.498 |  | 0.260 | 0.090-0.374 |
| 6A | 13,249 | 618.0 | 0.046 |  | 0.402 | 0.095-0.500 |  | 0.315 | 0.090-0.375 |
| 6B | 16,434 | 720.9 | 0.044 |  | 0.324 | 0.095-0.500 |  | 0.263 | 0.090-0.375 |
| 6D | 3,044 | 473.5 | 0.156 |  | 0.335 | 0.095-0.499 |  | 0.269 | 0.097-0.374 |
| 7A | 19,808 | 736.6 | 0.037 |  | 0.362 | 0.096-0.500 |  | 0.292 | 0.097-0.375 |
| 7B | 12,025 | 750.6 | 0.062 |  | 0.354 | 0.096-0.500 |  | 0.284 | 0.091-0.375 |
| 7D | 4,837 | 638.6 | 0.132 |  | 0.301 | 0.096-0.498 |  | 0.248 | 0.091-0.374 |
| A genome | 89,519 | 4934.5 | 0.099 |  | 0.365 | 0.095-0.500 |  | 0.291 | 0.090-0.380 |
| B genome | 146,270 | 5179.0 | 0.042 |  | 0.363 | 0.096-0.500 |  | 0.289 | 0.090-0.380 |
| D genome | 24,133 | 3950.4 | 0.202 |  | 0.340 | 0.096-0.498 |  | 0.275 | 0.090-0.370 |

^a^ Chr, Chromosome.

^b^ The physical map based on wheat genome sequences from the International Wheat Genome Sequencing Consortium (IWGSC, http://www.wheatgenome.org/).

^c^ PIC, Polymorphism information content.
