# Supplementary material for: Genome-wide association mapping of black point reaction in common wheat (Triticum aestivum L.)
Source: BMC Plant Biol. 2017 Nov 23;17:220. doi: 10.1186/s12870-017-1167-3 (PMC5701291; doi:10.1186/s12870-017-1167-3)
Supplement: Supplementary file 3 — LD decay estimated in 166 wheat accessions based on 12,324 markers from the 90 K and 660 K SNP arrays. (DOCX 97 kb) [file 12870_2017_1167_MOESM3_ESM.docx]

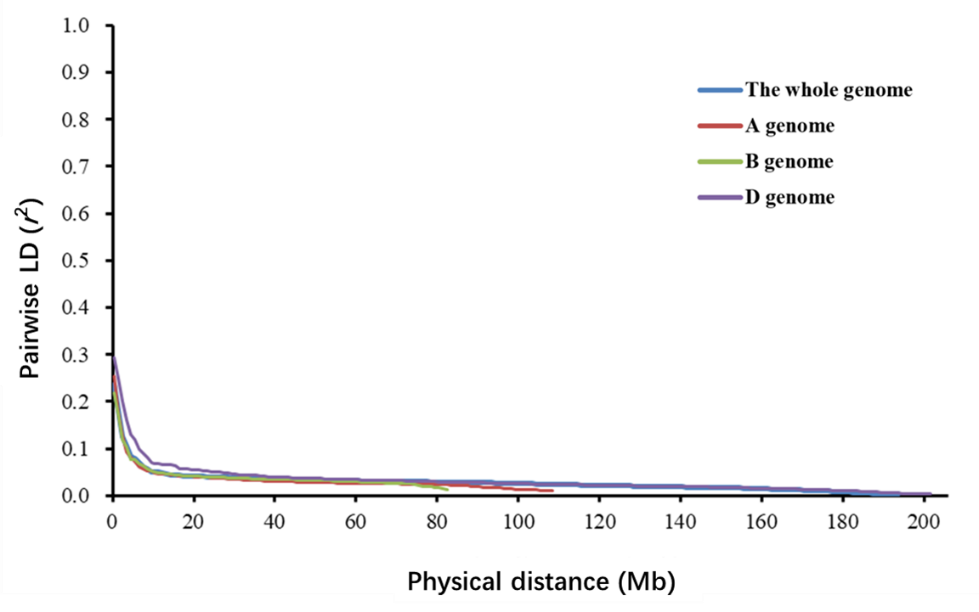


**Additional file 3: Figure S1** LD decay estimated for 166 wheat accessions based on 12,324 markers from the 90K and 660K SNP arrays.
