# Supplementary material for: Genome-wide association mapping of black point reaction in common wheat (Triticum aestivum L.)
Source: BMC Plant Biol. 2017 Nov 23;17:220. doi: 10.1186/s12870-017-1167-3 (PMC5701291; doi:10.1186/s12870-017-1167-3)
Supplement: Supplementary file 4 — Black point scores evaluated for 166 wheat accessions. Reported values are the best linear unbiased predictions (BLUP) value for black point scores across five environments. (DOCX 430 kb) [file 12870_2017_1167_MOESM4_ESM.docx]

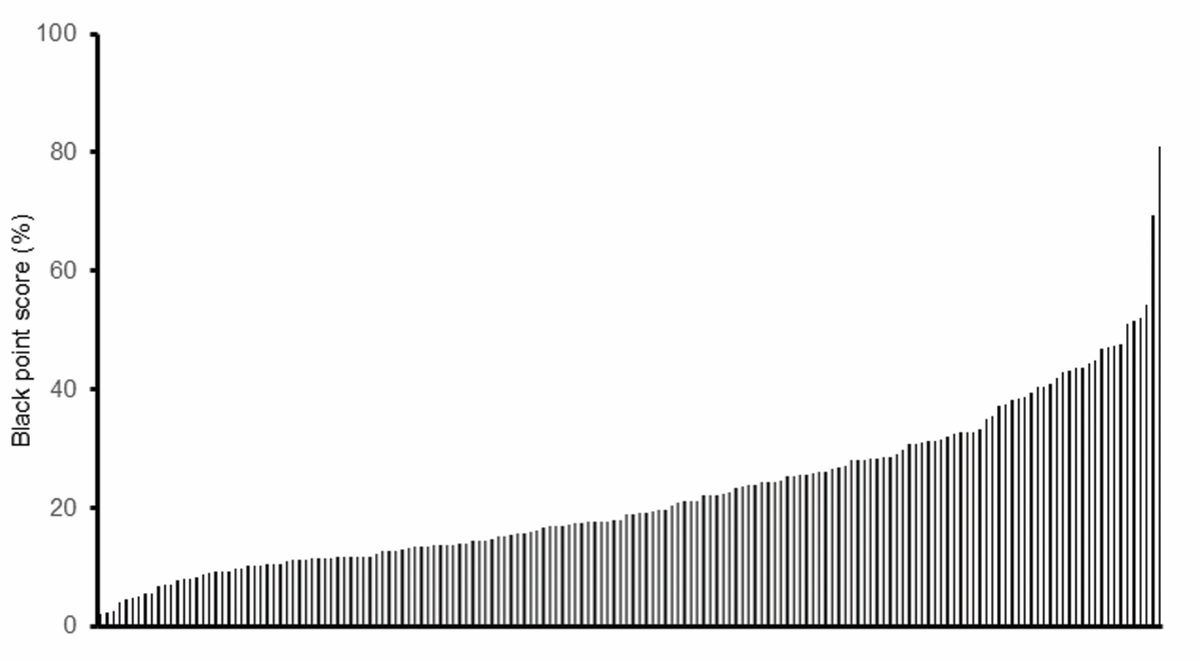


**Additional file 4: Figure S2** Black point scores evaluated in 166 wheat accessions. Reported values are best linear unbiased predictions (BLUP) value for black point scores across five environments.
