# Supplementary material for: Genome-wide association mapping of black point reaction in common wheat (Triticum aestivum L.)
Source: BMC Plant Biol. 2017 Nov 23;17:220. doi: 10.1186/s12870-017-1167-3 (PMC5701291; doi:10.1186/s12870-017-1167-3)
Supplement: Supplementary file 10 — Quantile-quantile (Q-Q) plot for black point scores in 166 wheat accessions by the mixed linear model (MLM) in Tassel v5.0. (a) Anyang 2013; (b) Anyang 2014; (c) Anyang 2015; (d) Suixi 2013; (e) Suixi 2014; (f) Best linear unbiased predictions (BLUP) value for black point scores across five environments. (DOCX 682 kb) [file 12870_2017_1167_MOESM10_ESM.docx]

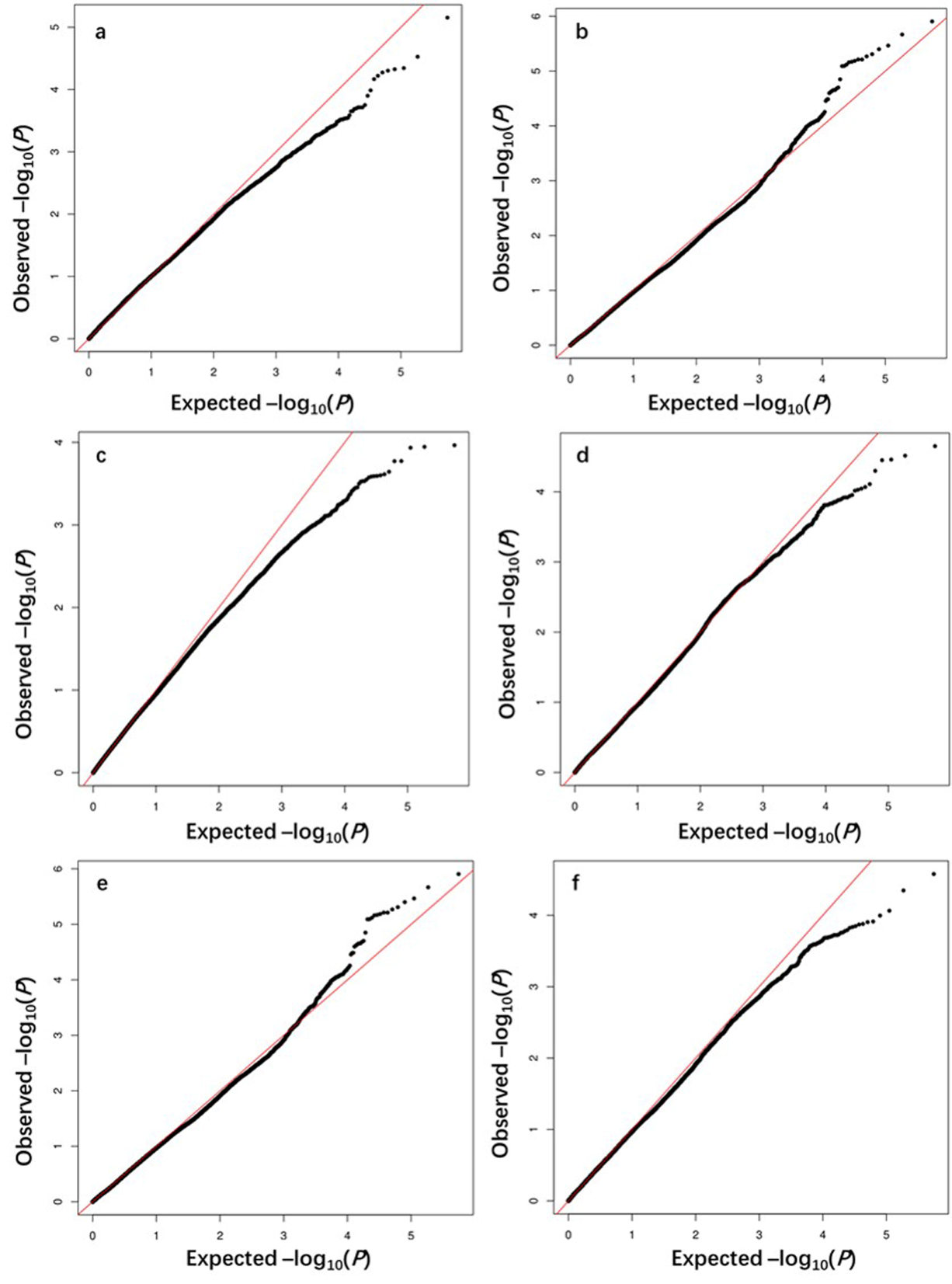


**Additional file 10: Figure S4.** Quantile-quantile (Q-Q) plot for black point scores in 166 wheat accessions by the mixed linear model (MLM) in Tassel v5.0. (a) Anyang 2013; (b) Anyang 2014; (c) Anyang 2015; (d) Suixi 2013; (e) Suixi 2014; (f) Best linear unbiased prediction (BLUP) values for black point scores across five environments.
