# Supplementary material for: Genome-wide association mapping of black point reaction in common wheat (Triticum aestivum L.)
Source: BMC Plant Biol. 2017 Nov 23;17:220. doi: 10.1186/s12870-017-1167-3 (PMC5701291; doi:10.1186/s12870-017-1167-3)
Supplement: Supplementary file 12 — LD heatmap of all wheat chromosomes showing extent of pairwise linkage disequilibrium between SNP markers significantly associated with black point reactions. (DOCX 4561 kb) [file 12870_2017_1167_MOESM12_ESM.docx]

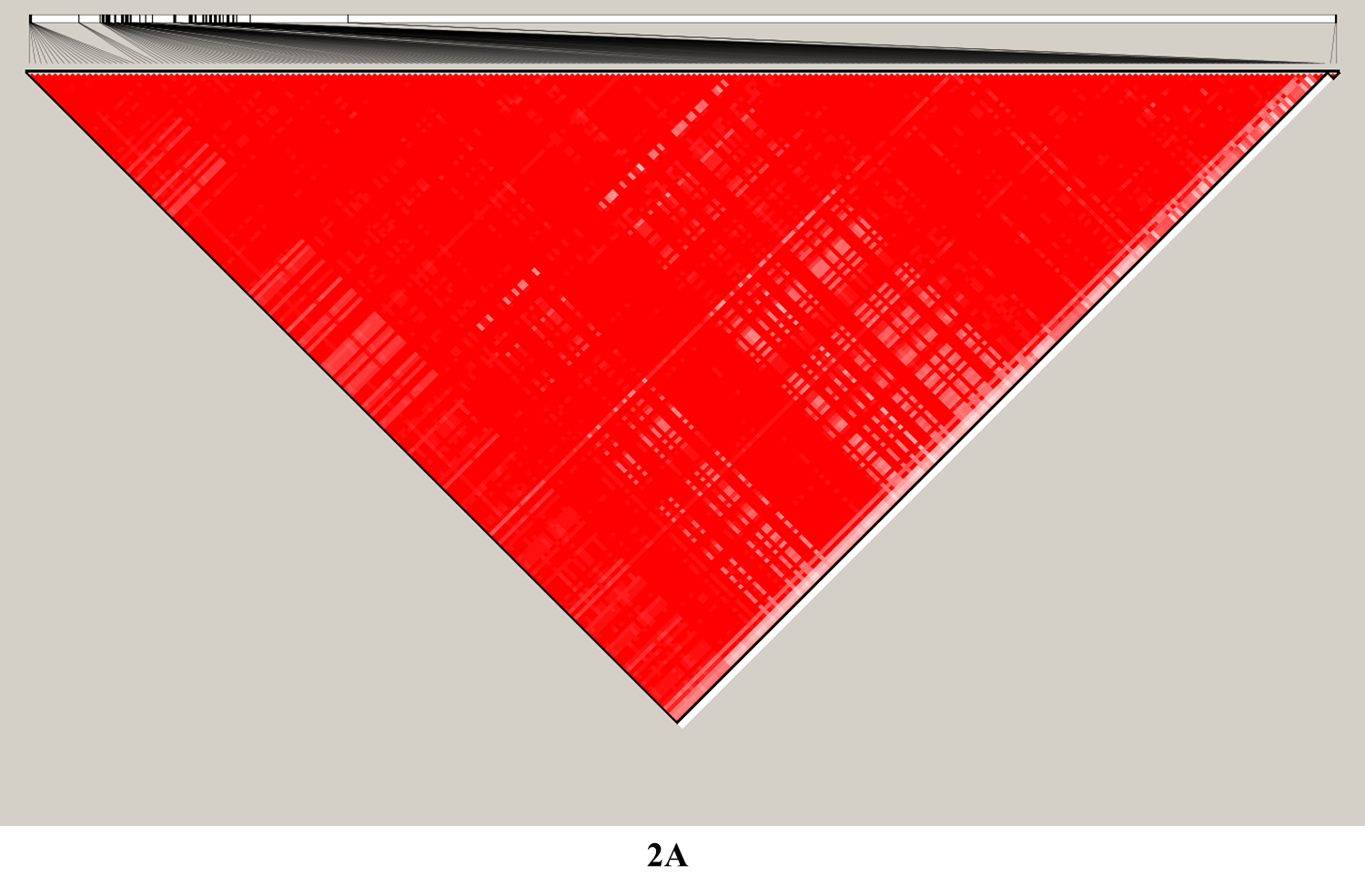


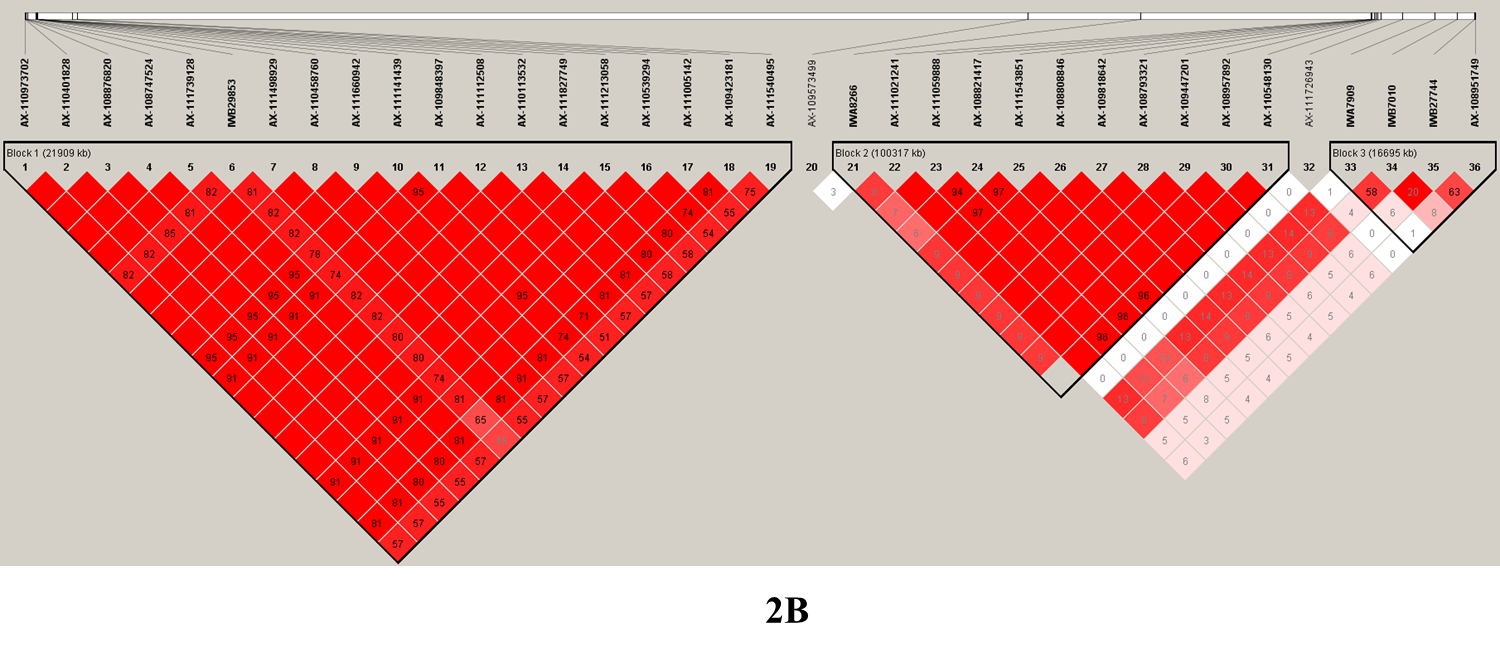


**Additional file 12: Figure S6** LD heatmap of all wheat chromosomes showing extent of pairwise linkage disequilibrium between SNP markers significantly associated with black point reaction.


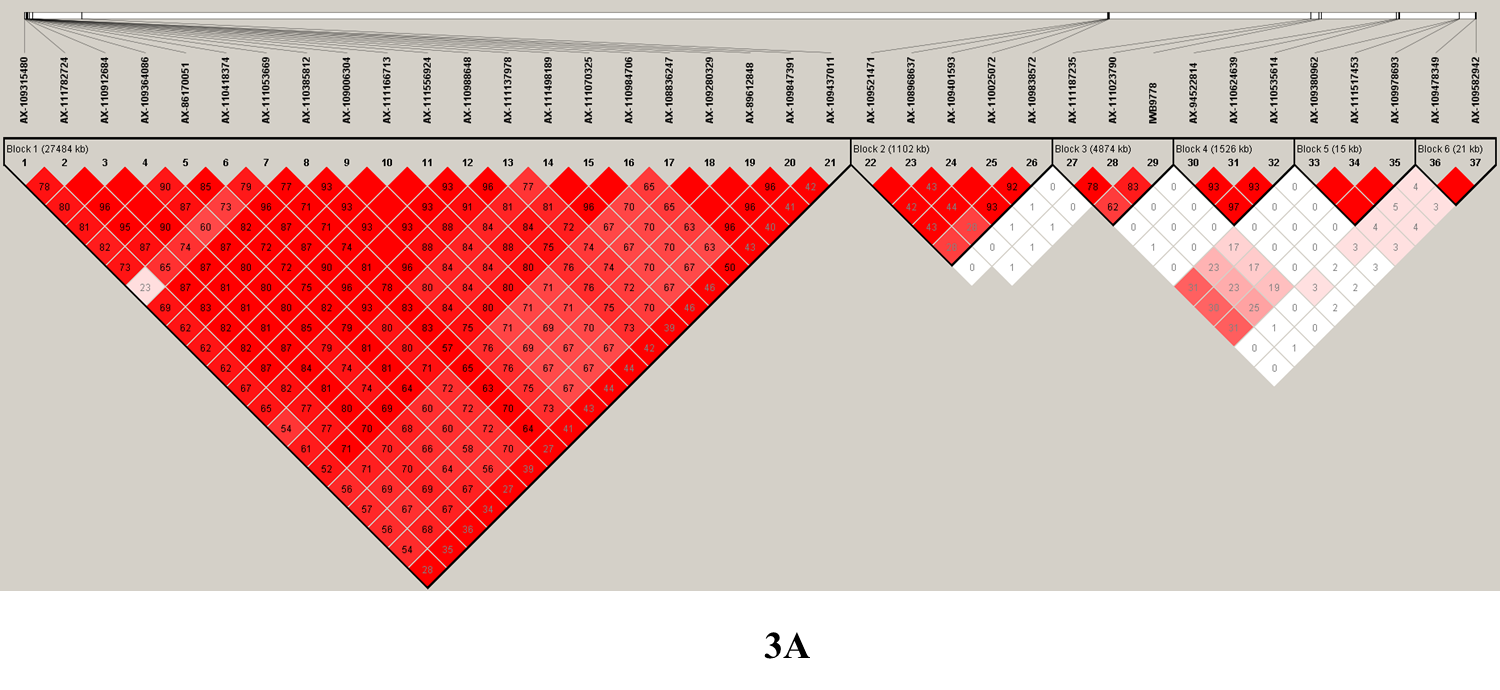


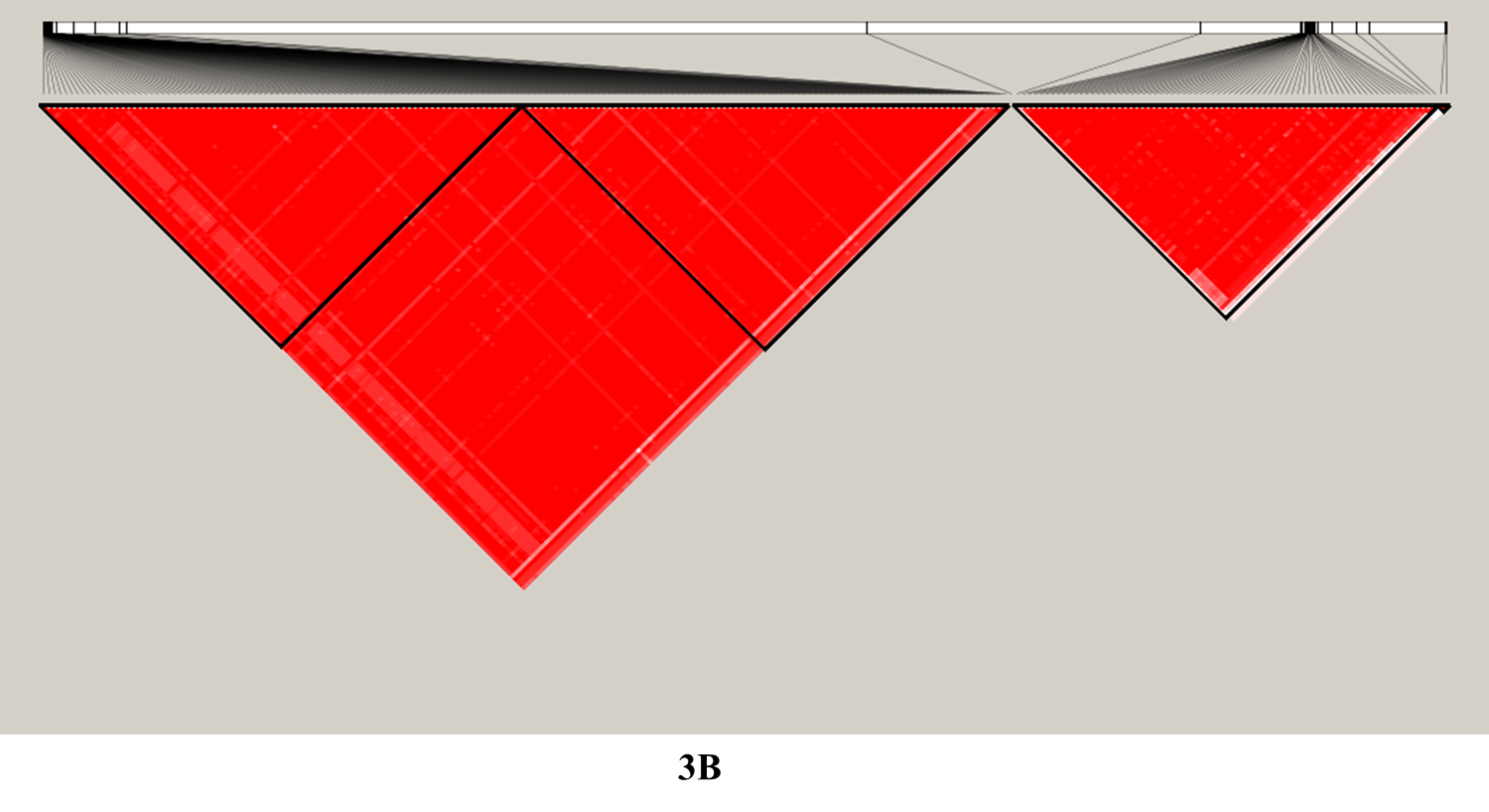


**Figure S6 continued**


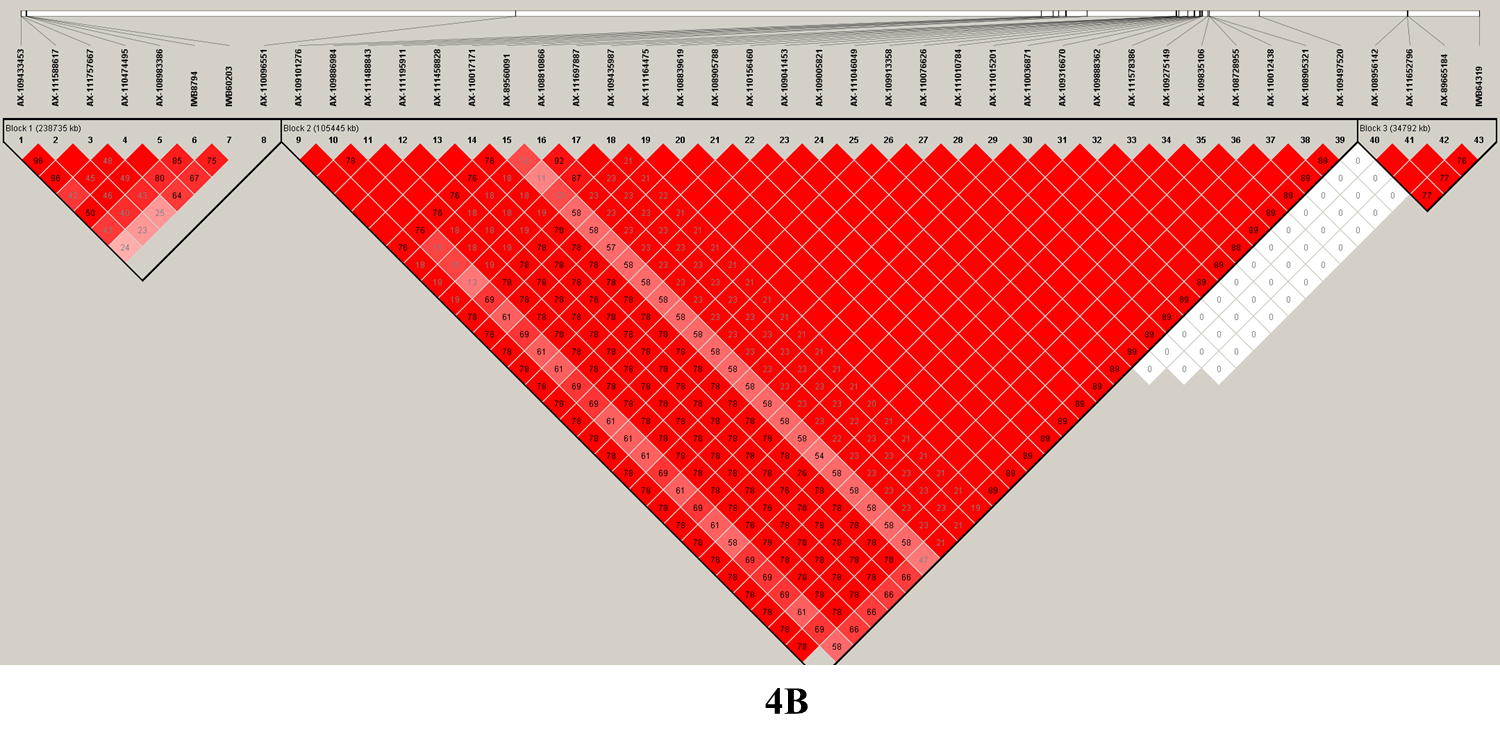


**Figure S6 continued**


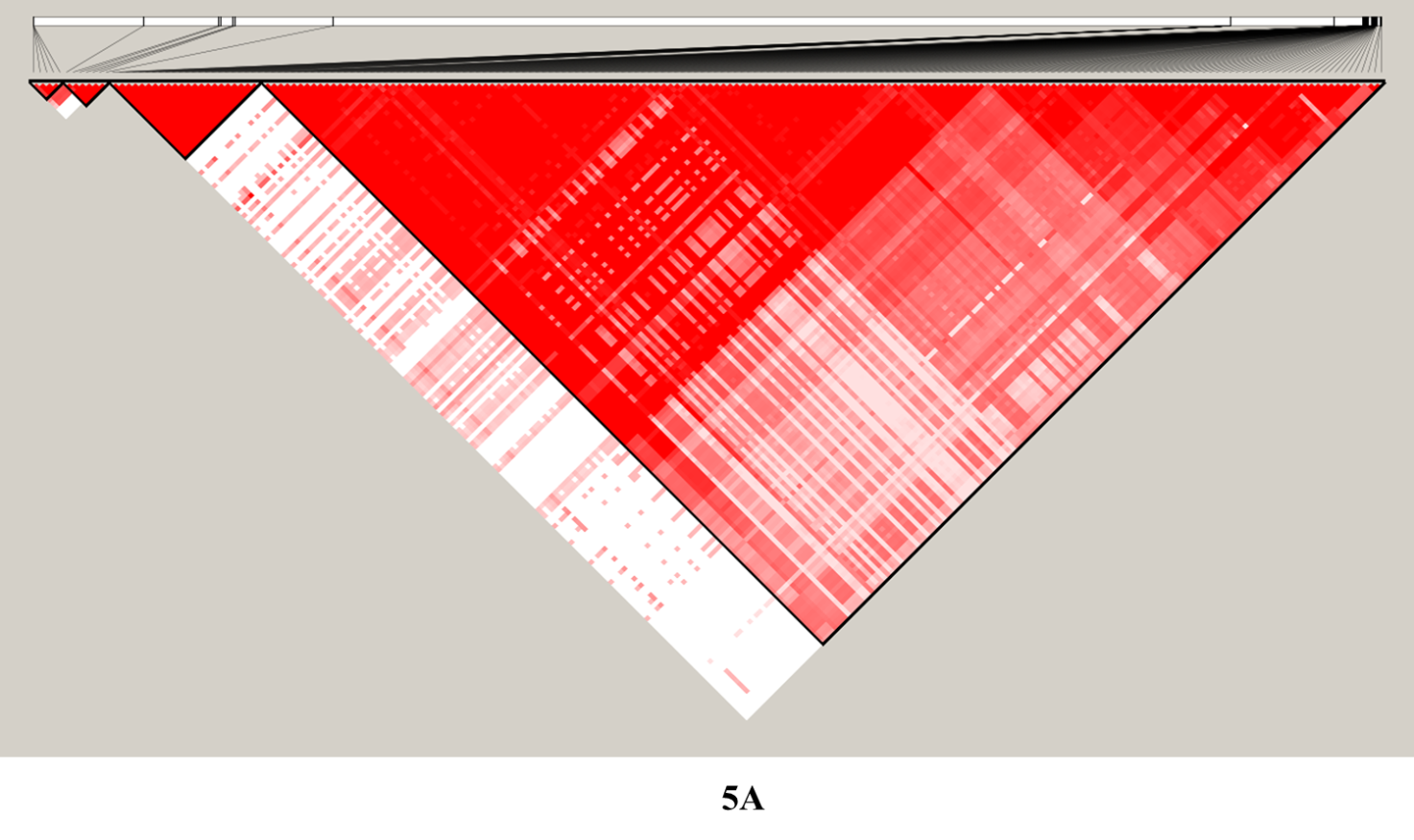


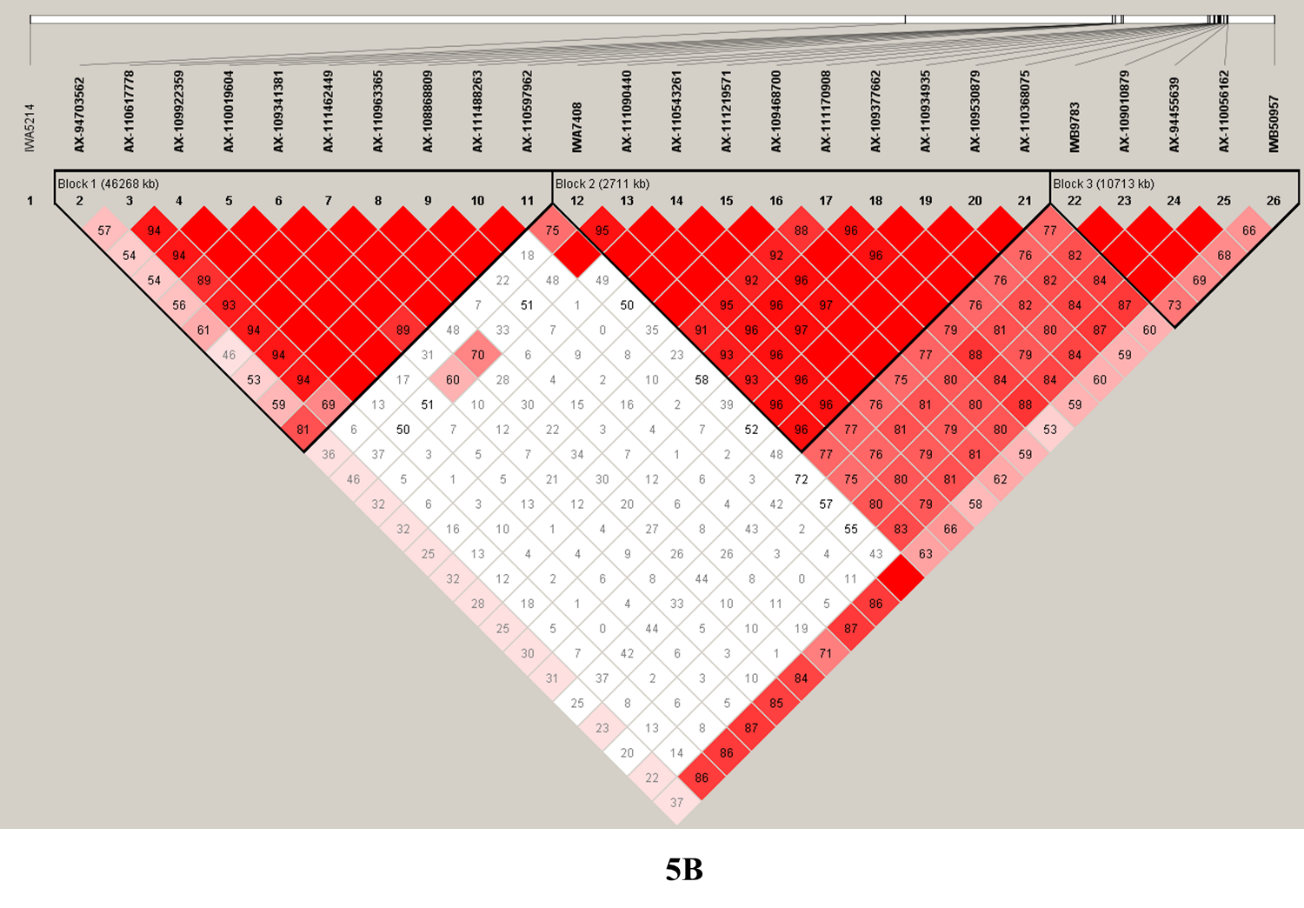


**Figure S6 continued**


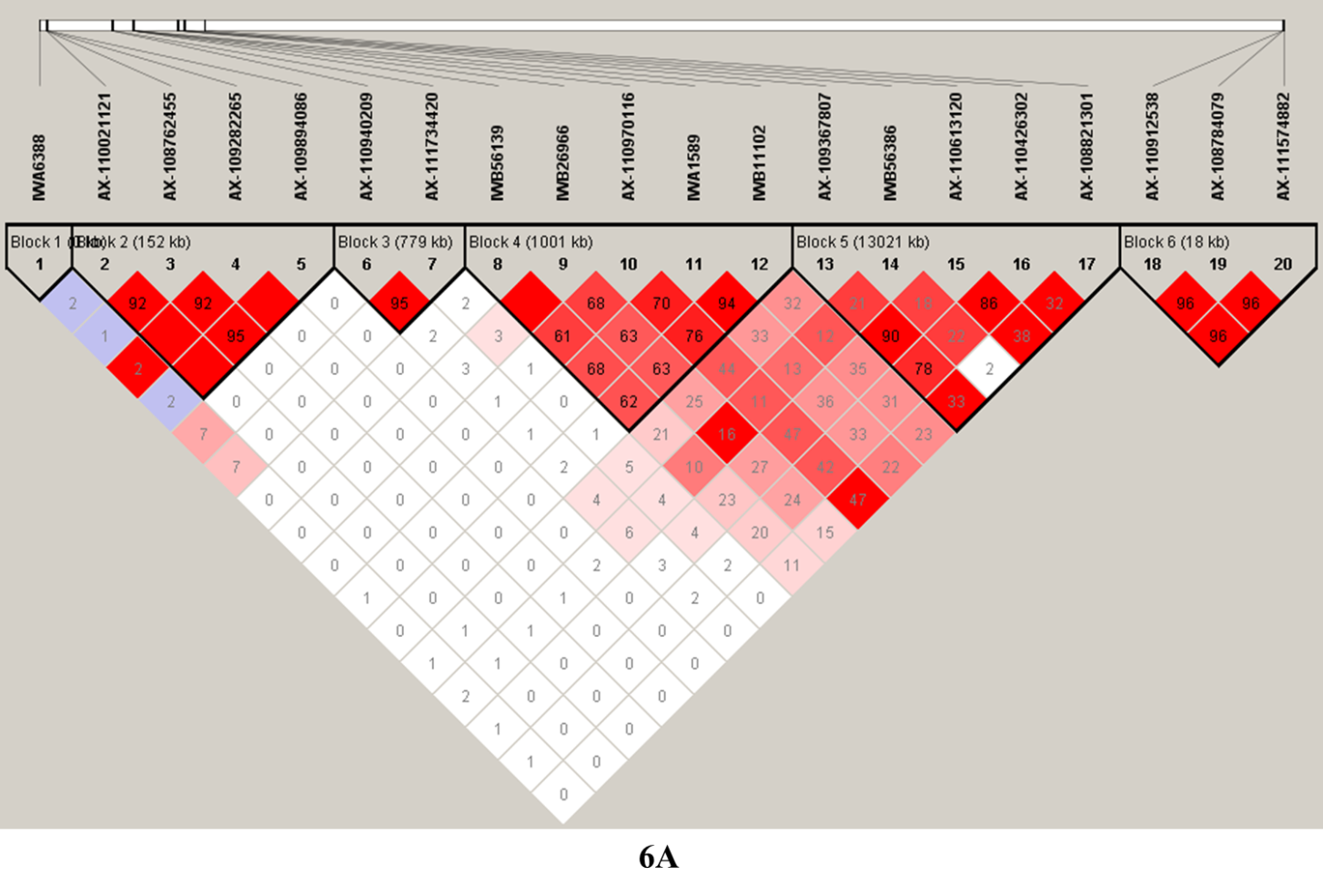


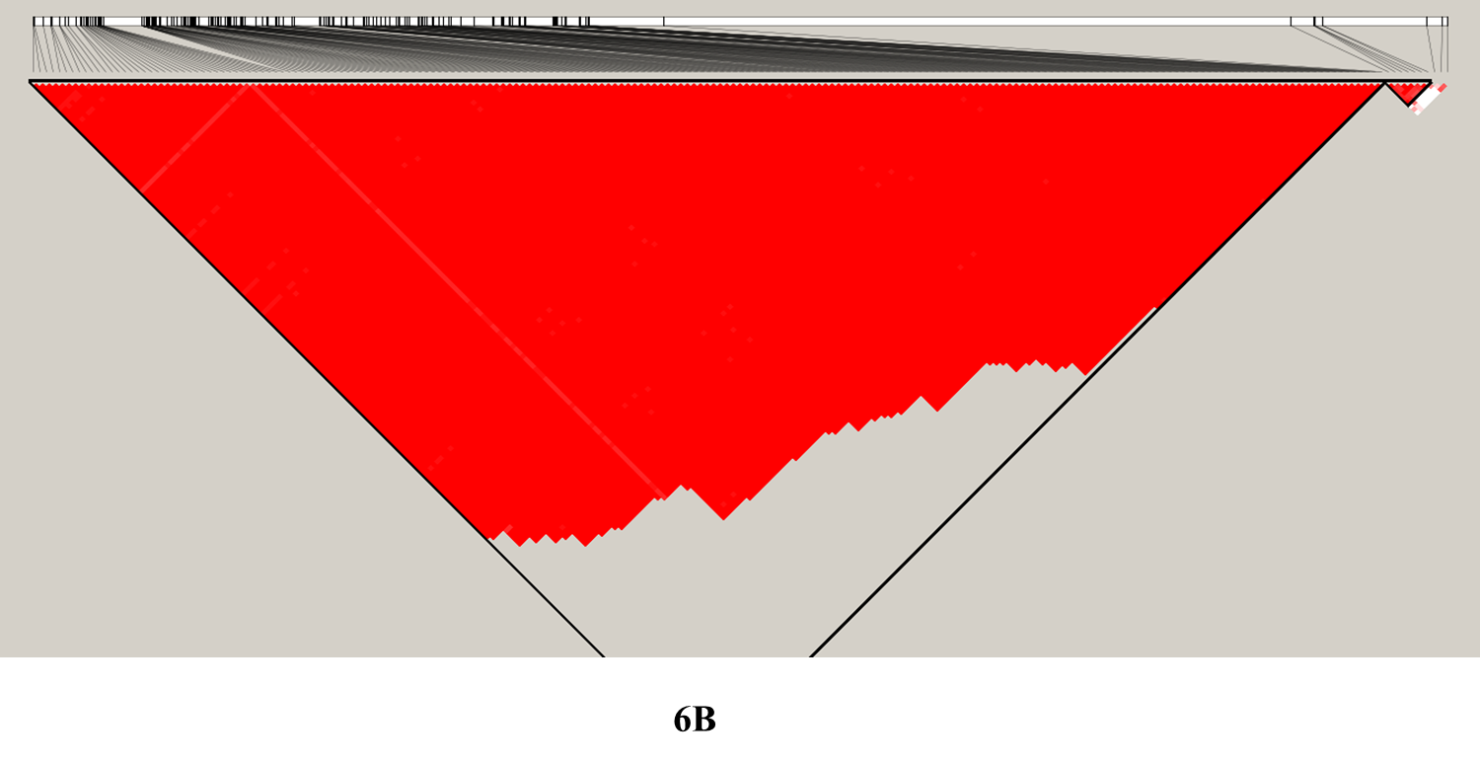


**Figure S6 continued**


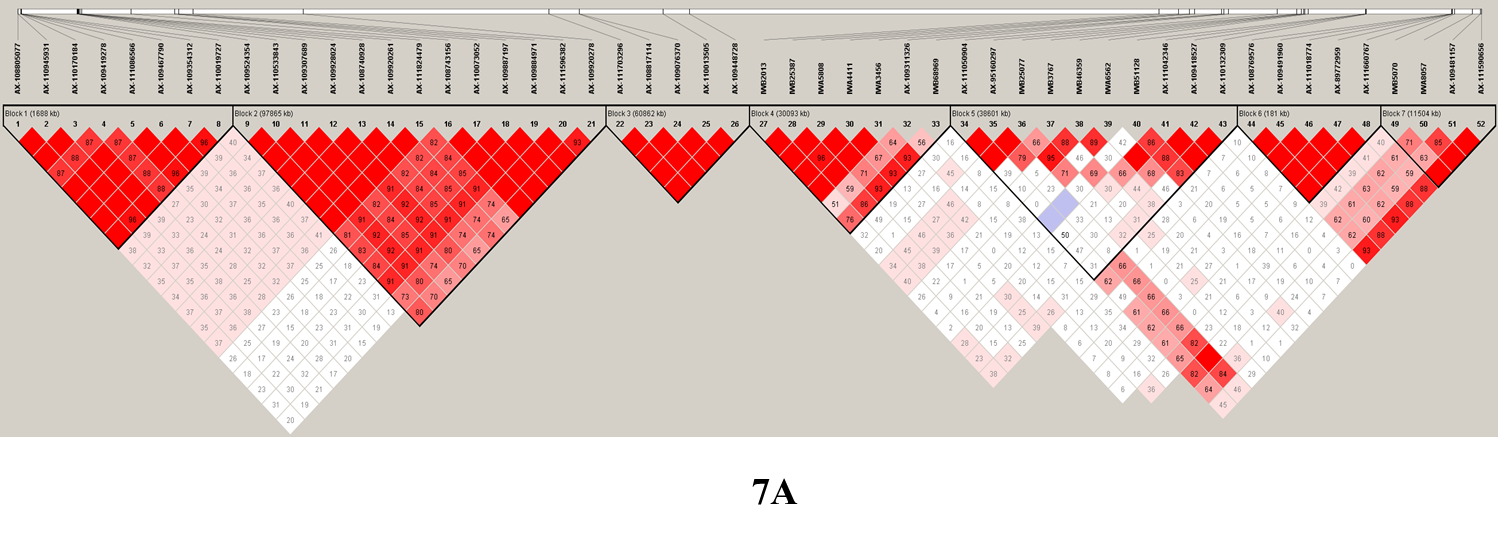


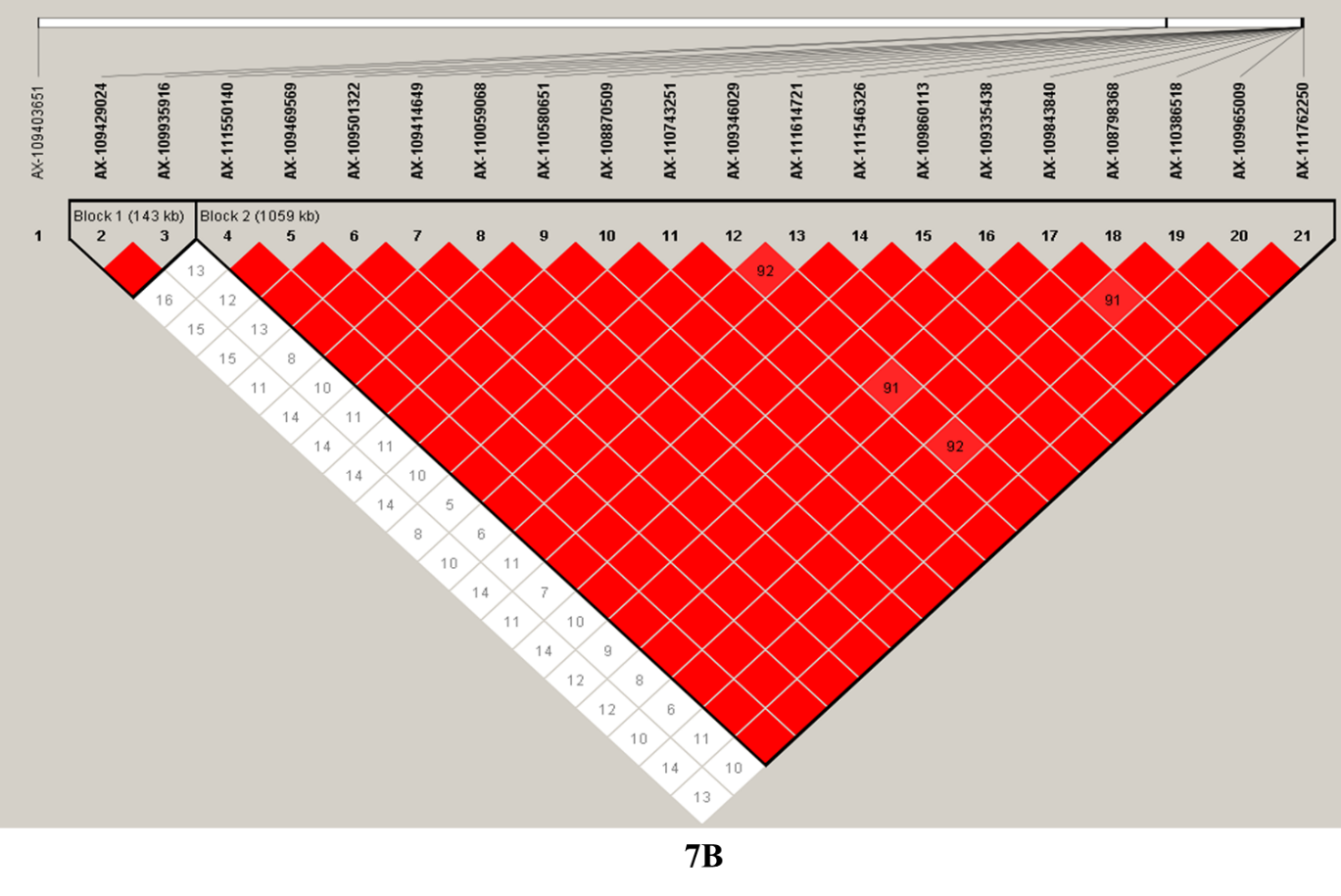


**Figure S6 continued**


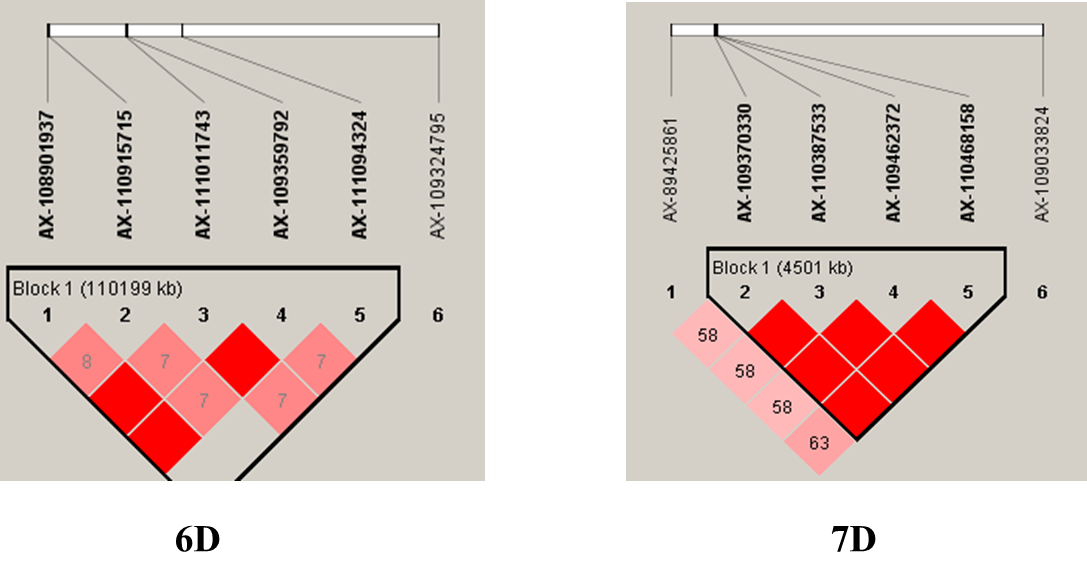


**Figure S6 continued**
