# Supplementary material for: Genome-wide association mapping of black point reaction in common wheat (Triticum aestivum L.)
Source: BMC Plant Biol. 2017 Nov 23;17:220. doi: 10.1186/s12870-017-1167-3 (PMC5701291; doi:10.1186/s12870-017-1167-3)
Supplement: Supplementary file 13 — Accessions with high black point resistance. (DOCX 17 kb) [file 12870_2017_1167_MOESM13_ESM.docx]

**Additional file 13: Table S7** Accessions with high black point resistance

| **Accession** | **No. of**  **favorable alleles** |  | **Black point score** | | | | | |
| --- | --- | --- | --- | --- | --- | --- | --- | --- |
|  |  |  | **E1 ^a^** | **E2** | **E3** | **E4** | **E5** | **BLUP** |
| Kitanokaori | 20 |  | 1.0 | 2.3 | 6.0 | 1.7 | 1.7 | 1.6 |
| Barra | 21 |  | 2.0 | 2.0 | 4.0 | 2.0 | 3.0 | 2.1 |
| Norin 67 | 18 |  | 3.3 | 2.0 | 5.0 | 1.5 | 4.0 | 2.1 |
| Nidera Baguette 20 | 21 |  | 2.3 | 4.3 | 8.5 | 1.3 | 3.0 | 5.6 |
| Xinong 979-005 | 20 |  | 2.3 | 3.0 | 9.5 | 3.3 | 4.0 | 6.3 |
| Klein Jabal1 | 21 |  | 1.7 | 7.7 | 11.5 | 2.3 | 4.3 | 3.6 |
| Yumai 21 | 21 |  | 7.7 | 2.3 | 8.0 | 2.5 | 4.7 | 6.1 |
| Klein Flecha | 18 |  | 1.7 | 7.7 | 11.5 | 2.3 | 4.3 | 7.8 |
| Shan 715 | 20 |  | 3.0 | 3.7 | 12.0 | 5.3 | 4.0 | 8.0 |
| Yannong 19 | 18 |  | 2.7 | 4.7 | 14.0 | 9.0 | 4.0 | 9.4 |
| Sagittario | 18 |  | 2.3 | 7.7 | 15.0 | 3.3 | 4.3 | 9.9 |
| Zhoumai 19 | 18 |  | 3.0 | 4.0 | 16.0 | 4.0 | 13.0 | 11.0 |
| Zhongmai 871 | 19 |  | 13.3 | 6.3 | 5.0 | 9.3 | 7.0 | 6.8 |
| Fu 936 | 21 |  | 10.0 | 5.0 | 6.5 | 11.0 | 8.7 | 7.6 |
| Zhongyu 5 | 18 |  | 10.7 | 8.3 | 11.5 | 9.0 | 5.7 | 9.7 |

^a^ E1: Anyang 2013; E2: Anyang 2014; E3: Anyang 2015; E4: Suixi 2013; E5: Suixi 2014; BLUP: Best linear unbiased prediction (BLUP) calculated from all five environments.
